# *At-CycD2* Enhances Accumulation of Above-Ground Biomass and Recombinant Proteins in Transgenic *Nicotiana benthamiana* Plants

**DOI:** 10.3389/fpls.2021.712438

**Published:** 2021-09-10

**Authors:** Lilya Kopertekh, Sven Reichardt

**Affiliations:** Institute for Biosafety in Plant Biotechnology, Julius Kühn-Institut (JKI) - Federal Research Centre for Cultivated Plants, Quedlinburg, Germany

**Keywords:** *At-CycD2*, *Nicotiana benthamiana*, plant architecture, recombinant proteins, transient expression

## Abstract

Transient expression in *Nicotiana benthamiana* holds great potential for recombinant protein manufacturing due to its advantages in terms of speed and yield compared to stably transformed plants. To continue improving the quantity of recombinant proteins the plant host will need to be modified at both plant and cellular levels. In attempt to increase leaf mass fraction, we transformed *N. benthamiana* with the *At-CycD2* gene, a positive regulator of the cell cycle. Phenotypic characterization of the T_1_ progeny plants revealed their accelerated above-ground biomass accumulation and enhanced rate of leaf initiation. In comparison to non-transgenic control the best performing line At-CycD2-15 provided 143 and 140% higher leaf and stem biomass fractions, respectively. The leaf area enlargement of the At-CycD2-15 genotype was associated with the increase of epidermal cell number compensated by slightly reduced cell size. The production capacity of the At-CycD2-15 transgenic line was superior to that of the non-transgenic *N. benthamiana*. The accumulation of transiently expressed GFP and scFv-TM43-E10 proteins per unit biomass was increased by 138.5 and 156.7%, respectively, compared to the wild type. With these results we demonstrate the potential of cell cycle regulator gene *At-CycD2* to modulate both plant phenotype and intracellular environment of *N. benthamiana* for enhanced recombinant protein yield.

## Introduction

The growing market for recombinant proteins requires effective and safe production platforms. Currently most of the recombinant proteins are manufactured in bacterial and mammalian cells, which are complex to handle and susceptible to contamination with human pathogens. The advances in molecular biology and plant biotechnology opened an avenue for using plants as bioreactors. Despite several advantages such as reduced upstream complexity, animal-free production, improved protein quality and speed in case of transient expression, improvements in protein yield, purification costs, and development of regulatory framework are essential to enable commercial utilization of plant-based production platform (Schillberg et al., 2019).

Two main approaches, namely stable transformation and transient expression, are currently used to manufacture foreign proteins in plant cells. Transient expression technology has made rapid and impressive progress in recent years and reached the status of established platform for commercial application (Lomonossoff and D’Aoust, 2016). This technique holds great potential for the production of rapid-response and emergency vaccines or biologics that was particularly shown for Ebola (The PREVAIL II Writing Group, for the Multi-National PREVAIL II Study Team, 2016), seasonal influenza (Ward et al., 2020), and COVID-19 (Capell et al., 2020; Diego-Martin et al., 2020) diseases.

Transient expression is a two-component system including expression vector and plant host. Among the expression vectors, virus vectors are very attractive for recombinant protein production due to the time efficiency, scalability and high yield of the target product. The majority of plant viral vectors used to date are based on RNA viruses, such as tobacco mosaic virus (TMV), potato virus X (PVX), and cowpea mosaic virus (CPMV; Hefferon, 2012). Two strategies, full virus strategy and deconstructed virus strategy, have been exploited for the construction of virus expression vectors. In the first strategy a wild type virus carries and expresses a gene of interest. In the second approach the limiting or undesirable for recombinant protein production viral functions are eliminated (Gleba et al., 2004; Peyret and Lomonossoff, 2015). Currently three deconstructed versions of TMV, which are deficient for the systemic virus movement, are available. These are magnICON® (Gleba et al., 2014), TRBO (Lindbo, 2007), and TMV launch vector (Musiychuk et al., 2007). In comparison to the three-component magnICON® system, one-component TRBO and TMV launch expression vectors may simplify the application. *Nicotiana benthamiana* is the plant of choice for transient expression of recombinant proteins (Powell, 2015). This plant belongs to *Solanaceae* family and originates from Australia. Its allotetraploid genome, which most probably results from hybridization of *Nicotiana sylvestris* and *Nicotiana tomentosiformis*, comprises 19 chromosomes (Goodin et al., 2008). A naturally occurring mutation in an RNA dependent RNA polymerase gene (Nb-RDR1) is, at least partly, responsible for *N. benthamiana*’s susceptibility to *Agrobacterium tumefaciens* and virus infection (Yang et al., 2004). Additional to this key trait further factors including its non-food status and easy cultivation make this species a dominant plant host for molecular farming. To date a number of biotechnology companies such as Medicago (Quebec, Canada), Kentucky Bioprocessing (Owensboro, KY, United States), iBio (Bryan, TX, United States), PlantForm (Ontario, Canada) and Leaf Expression Systems (Norwich, United Kingdom) use *N. benthamiana* as a plant host in their production systems (Bally et al., 2018). During the last decade significant efforts have been made in engineering *N. benthamiana* host including designing a supportive cell environment to improve the quality and quantity of recombinant proteins and modification of plant habitus to increase the space–time yield (Buyel et al., 2021). Diverse plant cell engineering approaches such as suppression of gene silencing (Arzola et al., 2011; Garabagi et al., 2012; Matsuo and Matsumura, 2017; Matsuo and Atsumi, 2019), reduction of unintended proteolysis (Jutras et al., 2020) and leaf proteome rebalancing (Robert et al., 2015) have proved to be effective in increasing foreign protein accumulation. Alteration of the plant habitus to increase biomass accumulation and subsequent recombinant protein yield can be established using two strategies, modulation of cultural practices (Fujiuchi et al., 2016) and modification of the host plant. In the first approach, optimization of light quality and planting density affected the accumulation of hemagglutinin (HA) in agroinfiltrated *N. benthamiana* (Fujiuchi et al., 2017; Shang et al., 2018). Low plant density provided 15–49% and 10–15% higher recombinant protein yield per unit harvested biomass and unit area-time, respectively (Fujiuchi et al., 2017). Another study demonstrated the impact of growth promoting hormone 6-Benzylaminopurine (6-BAP) and tip pruning on H1 vaccine antigen yield (Goulet et al., 2019). The apex pruning had negative effect on the total H1 yield, whereas treatment of plants with 6-BAP caused a 65–75% increase in the H1 accumulation. In the second strategy the genetic background of the host plant is modified using crossing or genetic engineering. For instance, the hybrid host *Nicotiana excelsiana*, which was developed by crossing *N. benthamiana* with *Nicotiana excelsior*, produces two times more biomass than *N. benthamiana* (Fitzmaurice, 2002). In the transgenic methodological approach, several classes of genes including those involved in photosynthetic pathways, hormone metabolism, transcription, signalling, secondary wall biosynthesis and cell cycle regulation may be useful in an attempt to increase the plant biomass production (Busov et al., 2008; Rojas et al., 2010; Lima et al., 2017). Among these genes, cell cycle regulators have already attracted considerable attention in order to alter the plant architecture.

The basic components of the plant cell cycle are G1 (postmitotic interphase), S-phase (DNA synthesis phase), G2 (premitotic interphase), and mitosis/cytokinesis. The cell cycle machinery is strongly regulated at two major checkpoints, G2/M and G1/S (Francis, 2007). The G2/M and G1/S transitions require the activity of cyclin dependent kinases (CDKs). CDK activity is orchestrated at multiple levels involving association with cyclins, interaction with inhibitory proteins and targeted proteolysis (Inze and De Veylder, 2006; Scofield et al., 2014). Down-regulation of multiple inhibitors of CDK activity, ICK genes (*ick1*/*ick2*/*ick6*/*ick7* and *ick1*/*ick2*/*ick5*/*ick6*/*ick7*) stimulated cell proliferation and resulted in larger organs and seeds in *Arabidopsis thaliana* (Cheng et al., 2013). In *Nicotiana tabacum* overexpression of the At-APC10 and At-CDC27a subunits of the anaphase–promoting complex (APC) controlling the transition of cell cycle phases in *A. thaliana* increased the plant biomass production (Rojas et al., 2009; Lima et al., 2013). In addition, combination of the *At-APC10* and *At-CDC27a* genes in *N. tabacum* by crossing resulted in synergistic effect compared to the parental lines containing individual subunits (Lima et al., 2013). Transformation of *N. tabacum* plants with the *CycD2* gene from *A. thaliana*, which is involved in controlling the transition of G1/S phase, promoted cell division and cell proliferation in leaf and cell meristem. The modified tobacco plants displayed normal cell and meristem size, but enhanced growth, increased rates of leaf initiation and accelerated development in all stages from seedlings to maturity (Cockcroft et al., 2000).

Here we demonstrate that stable expression of the *At-CycD2* gene in *N. benthamiana* resulted in the increased leaf and stem biomass accumulation in line At-CycD2-15 by 143 and 140%, respectively. The overall plant morphology of these transgenic plants was not affected. We also observed the enhanced transiently expressed foreign protein accumulation in the *At-CycD2* plants in comparison to non-transgenic *N. benthamiana*. The concentration of recombinant proteins increased from 2,496±689 to 3,457±401μg/g fresh leaf weight for GFP and from 620±195 to 971±308μg/g fresh leaf weight for scFv-TM43-E10 when wild type and At-CycD2-15 transgenic plants were compared. These results are relevant for the improvement of recombinant protein production per unit of biomass under contained conditions.

## Materials and Methods

### Plasmid Constructs

The pLH*-35S-At-CycD2* (Kopertekh and Schiemann, 2019) plant transformation vector and pJL-*TRBO-G* (TRBO-*gfp*) construct have been described previously (Lindbo, 2007). To generate TRBO-*scFv-TM43-E10* construct the *Not*I restricted pJL-*TRBO-G* plasmid was treated with T4 DNA polymerase, digested with *Pac*I and ligated with the scFv-TM43-E10 PCR product cut with *Pac*I-*Eco*RV. The PCR product was amplified from the pOPE101-*TM43-E10* template (Meyer et al., 2011) using the PacI-TM43-E10-forw and TM43-E10-EcoRV-rev primers and checked by sequence analysis. The PacI-TM43-E10-forw and TM43-E10-EcoRV primers are presented in Supplementary Table S1.

### Plant Transformation

The binary pLH-*35S-At-CycD2* vector was introduced into *A. tumefaciens* (recently renamed to *Rhizobium radiobacter*) strain LBA4404 by the freeze–thaw method (Holsters et al., 1978). Transformed colonies were selected on solid LB medium supplemented with 300mg/L streptomycin (Duchefa, Haarlem, Netherland), 100mg/L spectinomycin (Duchefa, Haarlem, Netherland), 50mg/L rifampicin (Duchefa, Haarlem, Netherland) and checked by PCR analysis. *A. tumefaciens*-mediated transformation of *N. benthamiana* leaf explants was performed as described previously (Kopertekh et al., 2004). Briefly, *N. benthamiana* leaves were removed from the 6weeks old plants and cut into 1cm squares. *A. tumefaciens* containing the pLH-*35S-At-CycD2* plasmid was cultured overnight at 28°C in LB medium with appropriate antibiotics. The cells were harvested by centrifugation and then resuspended in liquid MS medium (Duchefa, Haarlem, Netherland) to give an absorbency of 0.6 at 600nm. Leaf segments were immersed in the *A. tumefaciens* suspension for 20min, blotted dry on sterile paper, and placed on MS medium supplemented with 1mg/L 6-BAP (Duchefa, Haarlem, Netherland) and 0,1mg/L 1-naphthaleneacetic acid (NAA; Duchefa, Haarlem, Netherland). After 2days, explants were transferred to similar medium to which 500mg/L ticarcillin (Duchefa, Haarlem, Netherland) and 5mg/L phosphinothricin (PPT; Duchefa, Haarlem, Netherland) were added. Two weeks after cocultivation with *Agrobacterium*, the concentration of ticarcillin in the medium was decreased to 300mg/L. Shoots were excised and rooted on MS medium without plant growth regulators containing 300mg/L ticarcillin and 5mg/L PPT. Rooted plants were transferred to soil to set seeds.

For seed germination, T_1_ progeny seeds were surface-sterilised for 5min with 70% ethanol, rinsed 5 times with sterile water and germinated on solid MS medium supplemented with 20g/L sucrose, 0.5g/L 2-(N-morpholino)ethanesulfonic acid (MES; Roth, Karlsruhe, Germany) and 5mg/L PPT. After 2weeks, the number of PPT-resistant to PPT-sensitive plants was calculated to evaluate the segregation ratio. PPT resistant plants containing T-DNA of the pLH-*35S-At-CycD2* were transferred into the greenhouse and used in subsequent experiments. Plants and germinated seeds were kept in the controlled environment chamber or greenhouse at 24°C with 16day/8night photoperiod.

### Molecular Analysis of Transgenic Plants

To confirm the transgenic nature of the regenerated plants the genomic DNA was prepared from leaf material by the CTAB method and used as a template for the PCR reaction. PCR was performed with primer pairs that are specific to the *At-CycD2* and *bar* genes (Supplementary Table S1). Thermal cycling was done at 94°C for 5min followed by 30cycles at 94°C for 1min, 60°C for 1min, 72°C for 1min and 10min of a final elongation at 72°C. Reactions were performed using the PTC-200 Peltier Thermal Cycler (Bio-Rad, Feldkirchen, Germany).

To determine the number of T-DNA inserts, genomic DNA was extracted from leaf tissue of transgenic and non-transformed plants by DNeasy Plant Maxi Kit (Qiagen, Hilden, Germany). Southern blot analysis was performed with 16μg of DNA after restriction with *Eco*RI and *Hind*III. The restricted DNA was separated by 0.8% agarose gel and UV coupled to a Hybond N^+^ nylon membrane. Blots were hybridized with a *bar* probe, labelled with a PCR DIG Probe Synthesis Kit (Merck, Darmstadt, Germany). Supplementary Table S1 presents the *bar* primers used for generation of the DIG labelled *bar* probe. The membranes were developed and detected with a DIG Detection system (Merck, Darmstadt, Germany) using conditions suggested by the supplier.

RNA for the reverse transcription RT-PCR analysis was extracted using RNeasy Plant Mini Kit (Qiagen, Hilden, Germany). Total RNA (2μg) was reverse transcribed using the Maxima Reverse Transcriptase and random hexamer primer following manufacturer’s specifications (Thermo Scientific, Waltham, United States). RT-PCR was performed by the At-CycD2-349-forw, At-CycD2-rev and GAPDH-238-forw, GAPDH-238-rev primers specific to the *At-CycD2* and glyceraldehyde 3-phosphate dehydrogenase (*GAPDH*) genes, respectively (Supplementary Table S1).

### Phenotypic Evaluation of Transgenic Lines

To evaluate the phenotype of transgenic lines harbouring the *At-CycD2* gene T_1_ seeds were germinated on solid MS medium containing 5mg/L PPT, whereas wild type *N. benthamiana* seeds were germinated on non-selective medium. Non-transgenic and PPT resistant transgenic *N. benthamiana* seedlings were transferred to the greenhouse at 2weeks after sowing. Morphological data (plant height, number of leaves per plant, plant and stem biomass) were collected at 4weeks after planting. The experiment was repeated three times. Each replication included 15 plants per transgenic line or wild type non-transformed control. The unpaired T-test using SigmaStat software was used to determine differences among the mean values.

For the leaf area analysis, the second, third, fourth, fifth leaves from eight transgenic and eight wild type *N. benthamiana* plants were harvested at 4weeks after planting. Leaves were numbered from bottom to top. Leaf area was measured by ImageJ software^1^ after scanning. The data were statistically analysed with Mann–Whitney test using SigmaStat statistic software.

### Analysis of Cell Size and Cell Number

The cell size and number of cells for third leaf were compared in wild type and At-CycD2-15 transgenic plants. Only abaxial epidermal cells were considered in this study and they are referred to hereafter as epidermal cells throughout the text. The epidermal cells were first visualized using the GFP protein. To this end, fully expanded third leaves were agroinfiltrated with the pLH-*35S-gfp* construct at 4weeks after planting as described previously (Kopertekh and Schiemann, 2019). Three plants for each genotype were involved in this experiment. The leaves were collected 3days after agroinfiltration and scanned to calculate the leaf area as described in previous section. After this, the base, middle and tip zones of each leaf were randomly selected, investigated and photographed by a fluorescence microscope (Nikon Eclipse equipped with FITC filters). A total of 739 and 668 epidermal cells for the At-CycD2 and wild type genotypes, respectively, were drawn manually to calculate the cell area with ImageJ software. The distribution of the cell area per genotype was obtained by pooling data from all cells drawn for wild type and transgenic line. The mean epidermal cell number per leaf was calculated as the ratio of leaf area to mean of leaf epidermal cell area.

### Plant Agroinfiltration

*A. tumefaciens* strain AGL0 cultures containing the TRBO-*gfp* and TRBO-*scFv-TM43-E10* expression constructs were grown overnight at 28°C in LB medium supplemented with 50mg/L kanamycin (Duchefa, Haarlem, Netherland), pelleted by centrifugation and resuspended in MMA solution (10mM MES, pH 5.6, 10mM MgCl_2_, 150μM acetosyringone) to OD_600_ of 0.1. Middle leaves of 6weeks old *N. benthamiana* plants were infiltrated using a syringe without a needle.

For vacuum agroinfiltration the bacterial suspensions were prepared as described above. The *N. benthamiana* plants were immersed into the MMA infiltration solution and vacuum was applied for 1min at a vacuum pressure of 100–120 mBar and then slowly released. The vacuum system LVS 601 Tp (ILMVAC, Ilmenau, Germany) was used for the experiments.

The agroinfiltration experiments were repeated two times with 3–4 plants per replication. The probes for ELISA were harvested at 9days after agroinoculation (dpi). Each sample is a pooled sample from three middle leaves of one plant. The data were statistically analysed with Mann–Whitney test using SigmaStat statistic software.

### Quantitative ELISA

ELISA was used to quantify the amount of recombinant proteins (GFP, scFv-TM43-E10) in the protein extracts from the agroinfiltrated transgenic (line At-CycD2-15) and wild type *N. benthamiana* plants. Leaf material (200mg) was harvested, homogenized with motor and pestle in 2 volumes (w/v) of phosphate buffer (137mM NaCI, 2.7mM KCI, 10mM Na_2_HPO_4_, 1.8mM KH_2_PO_4_, pH 7.2) and clarified by centrifugation for 10min at 4°C. ELISA plates were coated overnight at 4°C with 100μl of fresh prepared plant extract. After incubation the plates were washed three 3 with PBS and blocked with PBS-B (PBS supplemented with 2% BSA) at room temperature for 2h. Following 3 washes with PBS the plates were probed with the anti-Myc (Sigma-Aldrich, Taufkirchen, Germany) and anti-GFP (Roche, Penzberg, Germany) antibody for scFv-TM43-E10 and GFP, respectively. Subsequently, the washing step was repeated and a goat anti-mouse IgG alkaline phosphatase conjugate (Sigma-Aldrich, Taufkirchen, Germany) in PBS-B was added and incubated at room temperature for 1h. Finally, the plates were developed for 30min at 37°C with the p-nitrophenyl phosphate as substrate after washing. Optical density was measured at 405nm in a SUNRISE ™ microplate reader (Tecan, Männedorf, Switzerland). All plates contained control proteins for a standard curve, which were diluted with PBS and processed as above.

## Results

### Generation and Verification of Transgenic Plants

**Figure 1:**
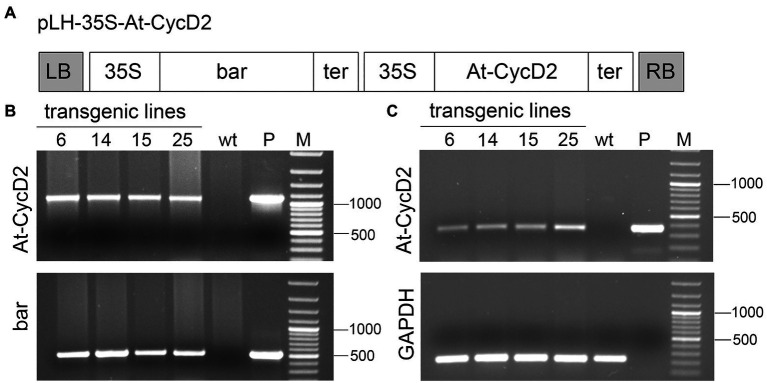
Molecular characterization of the *At-CycD2* transgenic lines. **(A)** Schematic representation of the pLH-*35S-At-CycD2* construct. The pLH-*35S-At-CycD2* plant transformation vector harbors *bar* and *At-CycD2* expression cassettes. In both expression units the constitutive expression of the *bar* and *At-CycD2* genes is regulated by the CaMV 35S promoter and terminator. Open boxes indicate the following genes: *bar*, *At-CycD2*, *bar* and *At-CycD2* genes, respectively; 35S, 35S promoter; ter, terminator. LB, RB, left and right border of T-DNA, respectively. **(B)** Verification of transgenic plants by PCR analysis. Genomic DNA from the PPT resistant regenerants (At-CycD2-6, At-CycD2-14, At-CycD2-15, and At-CycD2-25) was probed with primers specific to *bar* and *At-CycD2* genes. DNAs of wild type *N. benthamiana* plant (wt) and plasmid pLH-*35S-At-CycD2* (P) were included as negative and positive controls, respectively. GeneRuler 100bp Plus DNA marker (Thermo Scientific, Waltham, United States; M). **(C)** Expression analysis of the *At-CycD2* gene in transgenic lines. RNA isolated form the PCR-positive primary transgenic plants (At-CycD2-6, At-CycD2-14, At-CycD2-15, and At-CycD2-25) was subjected to RT-PCR analysis using *At-CycD2* and *GAPDH* specific primers. Non-transgenic *N. benthamiana* cDNA (wt) and plasmid pLH-*35S-At-CycD2* DNA (P) served as negative and positive controls, respectively. GeneRuler 100bp Plus DNA marker (Thermo Scientific, Waltham, United States; M).

To investigate the effect of the *At-CycD2* gene on *N. benthamiana* phenotype the pLH-*35S-At-CycD2* (Figure 1A) transformation vector containing *bar* and *At-CycD2* expression cassettes was introduced in the plant genome by *A. tumefacines*-mediated transformation. In the *At-CycD2* expression unit the *At-CycD2* gene is controlled by the 35S promoter and 35S terminator providing its constitutive expression.

A total of 21 regenerants were initially screened on selective medium containing 5mg/L PPT and subjected to PCR analysis using primers specific to *bar* and *At-CycD2* genes. The Bar-forw/Bar-rev primers produce a 503bp fragment of the *bar* gene, whereas the At-CycD2-forw/At-CycD2-rev primers amplify a 1,070bp fragment of the *At-CycD2* gene. For negative and positive controls, genomic DNAs from wild type plant and pLH-*35S-At-CycD2* plasmid, respectively, were taken as a template. The predicted PCR fragments were observed for 19 regenerants confirming the presence of the *bar* and *At-CycD2* sequences in investigated DNA samples. The results of the PCR analysis are shown in Figure 1B and Supplementary Figure S1.

The expression of the *At-CycD2* gene in PCR-positive T_0_ plants was investigated by the RT-PCR. CDNA synthesised from RNA of these plants was probed with the At-CycD2-349-forw/ At-CycD2-rev primers amplifying a 349bp PCR product of the *At-CycD2* gene and the GAPDH-238-forw and GAPDH-238-rev primers amplifying a 238bp fragment of endogenous gene GAPDH. Figure 1C presents RT-PCR data for At-CycD2-6, At-CycD2-14, At-CycD2-15 and At-CycD2-25 lines. This analysis showed that the *At-CycD2* gene was expressed in the investigated *N. benthamiana* lines.

*N. benthamiana* lines confirmed as transgenic at DNA and RNA level were transferred to the soil and allowed to set seeds. One regenerant did not grow in the soil. T_1_ progeny of At-CycD2 transgenic lines was subjected to segregation test. The observed ratio of PPT resistant to PPT sensitive seedlings suggests that all investigated lines are heterozygous transgenic events (Supplementary Table S2). Additionally, most of the investigated lines harbour multiple T-DNA insertion loci.

Seven transgenic lines, At-CycD2-1, At-CycD2-3, At-CycD2-4, At-CycD2-5, At-CycD2-10, At-CycD2-13, and CycD2-19 showed rare small chlorotic spots on some leaves and were excluded from the further analysis.

### Phenotypic Evaluation of the *At-CycD2* Transgenic Lines

**Figure 2:**
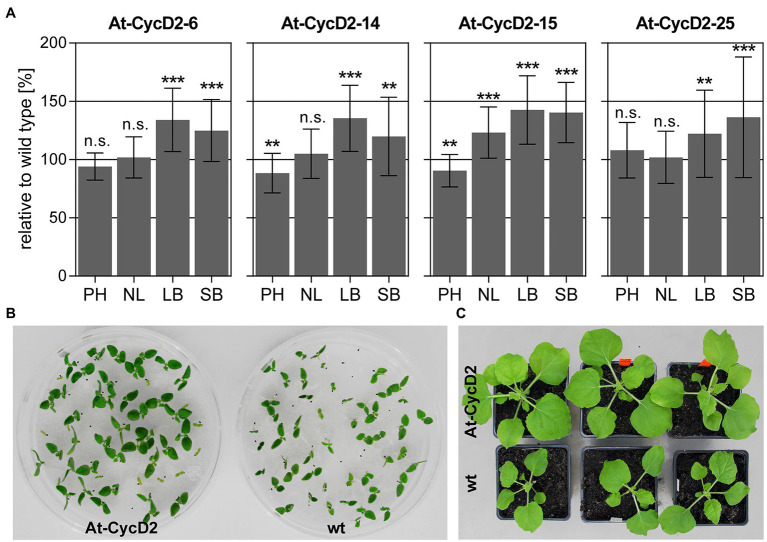
Phenotypic characterization of transgenic *N. benthamiana* lines expressing the *At-CycD2* gene. **(A)** Evaluation of plant height (PH), number of leaves (NL), leaf biomass (LB) and stem biomass (SB). Non-transgenic and T_1_ progeny of At-CycD2-6, At-CycD2-14, At-CycD2-15, and At-CycD2-25 transgenic plants were grown in greenhouse for 4weeks and assayed for the plant height, number of leaves and above ground biomass characteristics. The phenotypic parameters are expressed as a percent relative to the wild type. Values represent the means with standard deviation (*n*=45). Asterisks indicate significance as determined by the unpaired T-test, with ^**^ and ^***^ denoting *p*<0.01 and *p*<0.001, respectively. Not significant values are determined as ns. Non-transgenic and transgenic At-CycD2-15 plants grown *in vitro* **(B)** and in soil **(C)**. At-CycD2-15 and wild type *N. benthamiana* seeds were germinated *in vitro* on selective (5mg/L PPT) and non-selective MS medium, respectively, and photographed at 12days after sowing. The soil-grown plants were photographed at 21days after planting.

T_1_ seeds of 11 *N. benthamiana* transgenic lines expressing *At-CycD2* gene were germinated on selective MS medium, transferred to soil and grown in the greenhouse for 4weeks. Several morphological measurements such as plant height, number of leaves, leaf and stem biomass were conducted to evaluate the phenotype of these lines (Supplementary Table S3). Across the 11 investigated transgenic lines, we did not observe any phenotypic abnormalities. Transgenic events At-CycD2-6, At-CycD2-14, At-CycD2-15, and At-CycD2-25 showed an enhanced above ground biomass accumulation compared to wild type plants (Figure 2A; Supplementary Table S4). The relative leaf biomass accumulation varied from 122% (line At-CycD2-25) to 143% (line At-CycD2-15) and the differences between the control and transgenic genotypes were statistically significant. A statistical significant difference in relative stem biomass accumulation could be also observed for all 4 assessed lines. In comparison to wild type *N. benthamiana* plants the means for this parameter increased by 125, 120, 140 and 136% for the At-CycD2-6, At-CycD2-14, At-CycD2-15, and At-CycD2-25 transgenic lines, respectively. In terms of plant height lines At-CycD2-6 and At-CycD2-25 were similar to the non-transgenic control, whereas plants of lines At-CycD2-14 and At-CycD2-15 were 10–12% shorter than the wild type counterpart. Moreover, the enhanced leaf biomass accumulation for the At-CycD2-15 transgenic event was accompanied by an increased number of leaves by 123%. It should be mentioned that an enhanced initiation of secondary stem leaves also contributed to this phenotypic characteristic (Supplementary Figure S2).

The largest increase in biomass accumulation was observed for the At-CycD2-15 transgenic line. When compared to non-transgenic *N. benthamiana* the At-CycD2-15 line exhibited an enhanced growth of young seedlings *in vitro* and it was sustained during vegetative growth in soil (Figures 2B,C). Segregation test of T_1_ generation of this transgenic line showed a Mendelian ratio 3:1 of PPT resistant to PPT sensitive seedlings indicating the integration of the pLH-*35S-At-CycD2* T-DNA (T-DNAs) at a single locus (Supplementary Table S2). To further investigate the integration pattern of the At-CycD2-15 transgenic event, the genomic DNA from T_1_ progeny plants was digested with either *Eco*RI or *Hind*III and hybridized with the digoxigenin-labelled *bar* probe. Southern blot hybridization confirmed that one T-DNA insertion was integrated in a single locus in the At-CycD2-15 line (Supplementary Figure S3). We selected the At-CycD2-15 line for further investigation based on its genotypic and phenotypic characterization.

### Leaf Area and Cellular Size-Related Traits of the At-CycD2-15 Line

**Figure 3:**
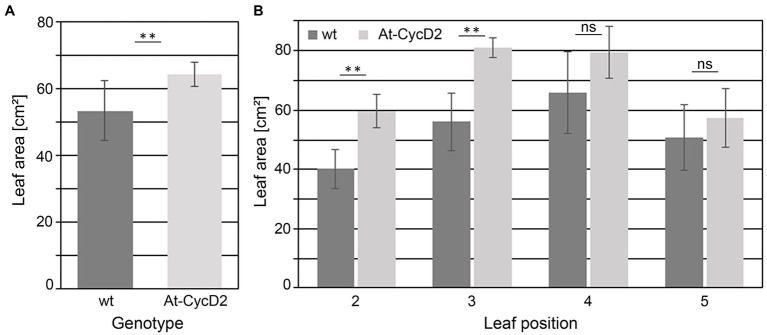
Leaf area of the At-CycD2-15 and wild type (wt) *N. benthamiana* plants. Leaves 2, 3, 4, and 5 from the soil-grown transgenic At-CycD2-15 and non-transgenic plants were harvested at 4weeks after planting to measure the leaf area. **(A)** Total leaf area. The leaf area for leaves 2, 3, 4, and 5 was measured and summarized for each plant. **(B)** Area of leaves at different positions. Values represent the means with standard deviation (*n*=8). Asterisks indicate significance as determined by the Mann–Whitney test, with ^**^ denoting *p*<0.01. Not significant values are determined as ns.

Phenotypic analysis of the At-CycD2-15 transgenic line revealed positive effect of the *At-CycD2* gene on leaf biomass production. Next, we asked whether this greater biomass accumulation is associated with the increased leaf size. To answer this question, the leaf area of the second, third, fourth, and fifth leaves from the greenhouse-grown plants of line At-CycD2-15 was measured at 4weeks after planting. In general, a beneficial impact of the At-CycD2 overexpression on leaf size was observed. The total leaf area for the second, third, fourth, and fifth leaves of the transgenic plants (64.5±3.6cm^2^) was significantly larger than that of the non-transgenic *N. benthamiana* plants (53.5±8.9cm^2^; Figure 3A). There was significant difference in the leaf area of the second leaf between the At-CycD2-15 transgenic line (59.8±5.7cm^2^) and the wild type (40.2±6.6cm^2^; Figure 3B). The leaf area of the third leaf was also significantly larger in the At-CycD2-15 transgenic line (81.1±3.3cm^2^) in comparison to the non-transgenic control (56.1±9.7cm^2^). The leaf area of the At-CycD2-15 fourth and fifth leaves was higher than that of the control, but the difference did not reach a significant level (Figure 3B).

**Figure 4:**
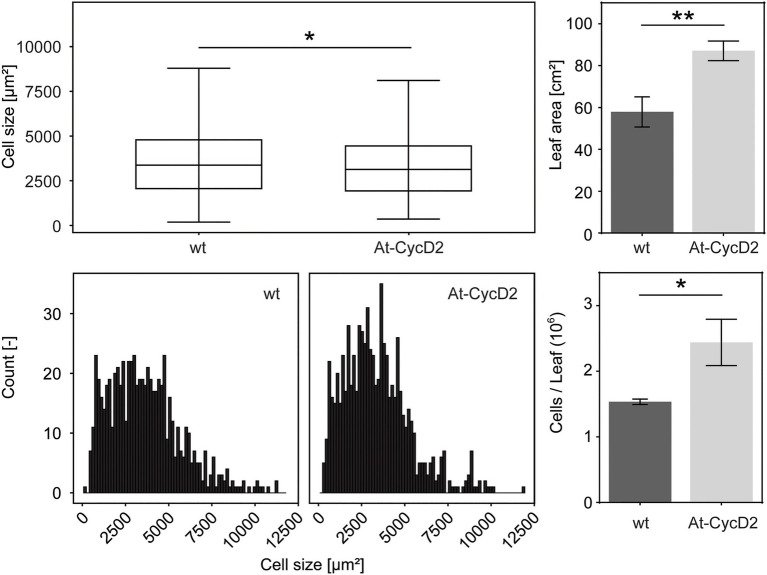
Comparison of leaf size-related characteristics for the At-CycD2-15 line and non-transgenic *N. benthamiana* plants. The third leaves for measurements were harvested from the wild type and At-CycD2-15 plants grown in soil at 4weeks after planting. The following parameters were evaluated: epidermal cell area **(A)**, cell distribution **(B)**, leaf area **(C)** and number of cells per leaf **(D)**. The number of total cells per leaf was calculated from **(C)** and **(A)**. The single cell distribution was obtained by pooling cell distributions observed for 3 plants. Values represent the means with standard error. Asterisks indicate significance as determined by the unpaired *T*-test, with ^*^ and ^**^ denoting *p*<0.05 and *p*<0.01, respectively.

The final size of plant leaves is determined by a combination of cell proliferation and cell expansion. To analyse the extent to which cell expansion and/or cell proliferation contribute to increased leaf size, we compared the area und number of epidermal cells in the At-CycD2-15 line and wild type *N. benthamiana*. We selected the third leaf for cellular analysis. The size of epidermal cells in the At-CycD2-15 transgene reached 3,384±71μm^2^ and was significantly smaller than the same parameter of the non-transgenic control (3,623±80μm^2^) at 4weeks after planting (Figure 4A). When the distribution of epidermal cell areas was considered, a larger proportion of expanding cells was found in the third leaf of wild type plants compared to the At-CycD2-15 transgene (Figure 4B). The calculation of the cell number revealed that overexpression of the *At-CycD2* gene in *N. benthamiana* led to a significant increase of the cell number (Figure 4C). The third leaves of the At-CycD2 transgenic line contained 2.44×10^6^ cells, whereas in wild type 1.54×10^6^ cells have been counted.

Taken together, our data indicate that the ectopic expression of the *At-CycD2* gene in *N. benthamiana* resulted in larger leaves primarily due to an increased cell number.

### Production of Recombinant Proteins in At-CycD2-15 Transgenic Plants

**Figure 5:**
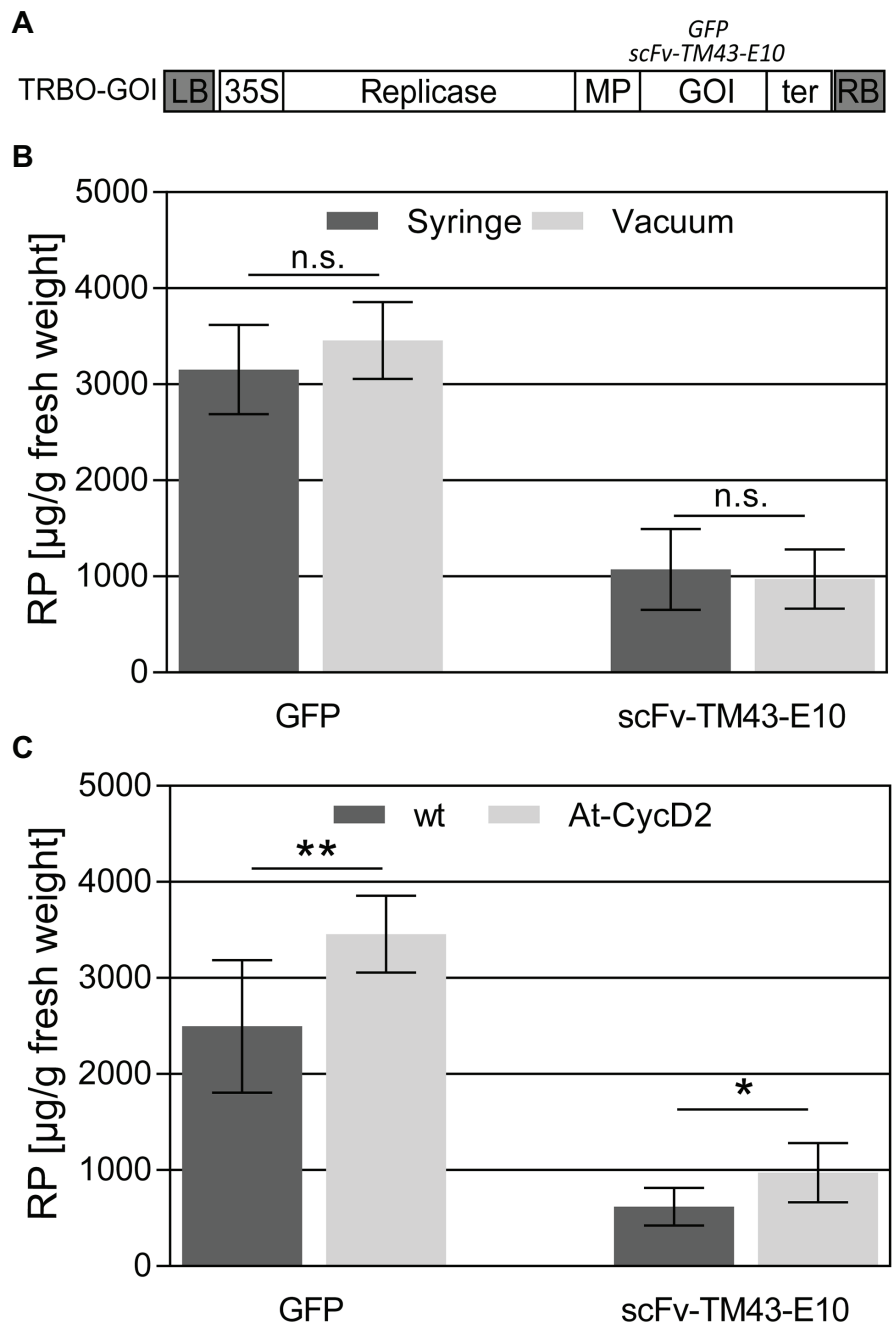
Accumulation of recombinant proteins in leaves of *N. benthamiana*. **(A)** Schematic representation of the TRBO expression vector. Open boxes indicate the following genes: Rep, viral replicase; MP, movement protein; GOI, gene of interest; 35S, 35S promoter. LB, RB, left and right border of T-DNA, respectively. Wild type (wt) and At-CycD2 transgenic plants were agroinfiltrated with the TRBO-*gfp* and TRBO-*scFv-TM43-E10*. **(B)** Comparison of the agroinfiltration methods (vacuum and syringe) in the At-CycD2-15 transgenic plants. **(C)** Comparison of the GFP and scFv-TM43-E10 accumulation in vacuum infiltrated wild type (wt) and At-CycD2-15 transgenic plants. Recombinant protein concentration is expressed in μg/g leaf fresh weight. Y-axis, RP is a recombinant protein. Values represent the means with standard deviation (*n*=7). Asterisks indicate significance as determined by the Mann–Whitney test, with ^*^ and ^**^ denoting *p*<0.05 and *p*<0.01, respectively. Not significant values are determined as ns.

To investigate the production capacity of the At-CycD2-15 transgenic line wild type and At-CycD2-15 transgenic plants were inoculated with the TMV-based deconstructed TRBO expression vector carrying the *gfp* and *scFv-TM43-E10* genes (Figure 5A) and evaluated for the recombinant protein yield at 9dpi. In the first set of experiments the efficiency of syringe and vacuum infiltration methods in the At-CycD2-15 transgenic plants was compared. The accumulation of the GFP and scFv-TM43-E10 proteins was estimated by ELISA. As shown in Figure 5B no significant differences between the vacuum and syringe infiltrated samples were observed for both the GFP and scFv-TM43-E10 proteins. In order to assess the production capacity of the At-CycD2-15 genotype, the accumulation of the GFP and scFv-TM43-E10 proteins was quantified in non-transgenic and At-CycD2-15 transgenic plants following a vacuum infiltration procedure (Figure 5C). Infiltration of wild type and At-CycD2-15 transgenic plants with the TRBO-GFP expression vector led to the production of 2,496±689μg/g fresh leaf weight (FW) and 3,457±401μg GFP /g fresh weight (FW), respectively. The scFv-TM43-E10 accumulation levels reached 620±195μg/g FW and 971±308μg/g FW in non-transgenic and At-CycD2-15 transgenic plants, respectively. Therefore, the presence of the *At-CycD2* gene enhanced the accumulation of both recombinant proteins.

## Discussion

In this study, the stable genetic transformation with the *At-CycD2* gene from *A. thaliana* was used to enhance the *N. benthamiana* biomass accumulation. The *At-CycD2* gene is a member of the D-type cyclin family, which is involved in the signal transduction pathway mediating cell cycle progression (Soni et al., 1995). This pathway involves the activation of the CDKs by association with cyclin(s), CDK-mediated inhibition of the retinoblastoma-related protein (RBR) which regulates the transcription factor E2F promoting expression of the S-phase genes (Inze and De Veylder, 2006). In the CDK complex, the cyclin determines the choice of substrate protein whereas the catalytic CDK subunit phosphorylates protein (Wittenberg, 2005). Immunoprecipitation experiments carried out by Cockcroft et al. (2000) demonstrated that the At-CycD2 interacted with the *N. tabacum* CDC2a protein to form functional CDK complex.

We established 19 At-CycD2 lines, verified as transgenic by PCR and segregation analysis. Ectopic expression of the *At-CycD2* gene led to enhanced stem and leaf biomass accumulation in four transgenic lines (At-CycD2-6, At-CycD2-14, At-CycD2-15, and At-CycD2-25). These transgenic lines exhibited normal morphology at all stages of the development. Our results are in agreement with those reported previously. Overexpression of the *At-CycD2* gene in *N. tabacum* resulted in lines with accelerated growth rate, observed as an increased rate of the above-ground biomass accumulation, when compared with the non-transgenic control (Cockcroft et al., 2000). This faster growth is a consequence of both shorter cell cycles and a higher growth fraction in meristematic cells, whereas the meristem size was unaffected (Boucheron et al., 2005). In another study, which investigated the regulation of cell division in stem tissue of the At-CycD2 expressing tobacco plants, enhanced cell division rates in vascular cambium and increased secondary xylem differentiation have been observed (Fujii et al., 2012). In contrast to our study an adverse effect of the *At-CycD2* gene on *N. tabacum* phenotype has not been reported by Cockcroft et al. (2000).

Two mechanisms may be proposed to explain the effect of the At-CycD2 on the plant phenotype. First, the At-CycD2-CDK complex could accelerate G1 phase resulting in an increased rate of cell division in meristematic tissue. A further hypothesis could be the involvement of the At-CycD2-CDK in cellular growth within the meristem due to the effect on general biosynthesis. As a consequence of this shorter time could be required to reach a critical cell size during G1 phase leading to cell division (Cockcroft et al., 2000; Boucheron et al., 2005).

We selected the At-CycD2-15 line due to its genotypic (single locus T-DNA integration pattern) and phenotypic (increased biomass accumulation) characteristics. The modified phenotype of the At-CycD2-15 line was associated with a total larger leaf area. Leaf area is affected by both genetic and environmental factors (Cookson et al., 2005). When plants are grown in stable environmental conditions final leaf area at a given rank on the plant is related to the epidermal cell number (Tisné et al., 2008). In accordance to this view of leaf development we observed the increased cell number in the third leaf of the At-CycD2-15 transgenic line in comparison to the wild type control. This increase in the cell number was accompanied by a decrease in the cell area. This compensatory effect between cell number and cell area has been reported for several cell cycle regulator genes. For example, for the *At-CycD3*gene an increased number of smaller cells have been observed in leaves of transgenic *A. thaliana* in comparison to the non-transgenic plants (Dewitte et al., 2003). In contrast in transgenic *A. thaliana* lines overexpressing Kip-related proteins (KRPs), which inhibit cell cycle progression *via* downregulation of the CDK activity, a reduced cell number was escorted by an increase in cell area (De Veylder et al., 2001).

The At-CycD2-15 transgenic line developed in this study is of particular interest for molecular farming due to its increased leaf mass fraction and genetic background supporting enhanced recombinant protein production. The combination of stably integrated *At-CycD2* gene with the transiently provided *gfp* and *scFv-TM43-E10* genes resulted in enhanced accumulation of the GFP and scFv-TM43-E10 proteins. In our previous study we demonstrated the beneficial effect of the At-CycD2 on the expression of the recombinant proteins expressed by the full TMV-based vector in a transient assay (Kopertekh and Schiemann, 2019). Co-infiltration of TMV-gfp, TMV-scFv-TM43-E10, and pLH-*35S-At-CycD2* vectors resulted in 3 and 2 fold increase in the accumulation of GFP and scFv-TM43-E10 recombinant proteins, respectively. The present investigation extends this finding and shows the influence of the *At-CycD2* cell cycle regulator gene, which was stably integrated in the *N. benthamiana* genome, on the production of foreign proteins expressed by the deconstructed TMV-based vector TRBO. Another study showed that virus-derived cell cycle regulators, the *RepA* genes from tobacco yellow dwarf virus and maize streak virus, the *Clink* gene from banana bunchy top virus, and the *REn* gene from tomato leaf curl virus, co-expressed with the pEAQ-GUS vector significantly increased the GUS accumulation levels about 2 to 3 fold (Norkunas et al., 2018). Both investigations provide data indicating that plant and virus derived cell cycle regulator genes have a positive impact on foreign protein accumulation in *N. benthamiana*.

In contrast to DNA viruses, there are only few publications, which are devoted to the interaction of TMV and host cell cycle machinery. For instance, early investigations of the TMV accumulation in *N. sylvestris* protoplasts revealed that the host cell position in the cell cycle impact the attachment efficiency of TMV (Gould et al., 1981). Investigating the function of the cell division cycle protein 48 (CDC48) from *A. thaliana* during TMV infection Niehl *et al.* proposed a model in which CDC48 participates in the regulation of TMV replication and cell-to-cell transport *via* interaction with the TMV movement protein (Niehl et al., 2012, 2013). In the At-CycD2/TMV combination transient application of the *At-CycD2* cell cycle regulator gene increased the virus RNA accumulation (Kopertekh and Schiemann, 2019). Although the mechanism of this phenomenon remains to be investigated the modulation of virus replication and/or cell-to-cell movement in the presence of the *At-CycD2* may be proposed.

The efficiency of transient expression in *N. benthamiana* depends on 2 yield-related quantities, recombinant protein accumulation per unit harvested biomass and leaf biomass per plant before agroinfiltration (Schillberg et al., 2019). The modified phenotype of the At-CycD2-15 transgenic line characterized by a significant increase in the leaf mass fraction contributes to the recombinant protein yield per unit of biomass. Application of this line can reduce the plant density during upstream production. The positive impact of reduced plant density during upstream production has been reported for the recombinant HA. The whole shoots from the plants, which were grown at low plant density, provided 15–49% higher yield per unit harvested biomass and 10–15% higher yield per unit area-time (Fujiuchi et al., 2017). The second phenotypic trait of the At-CycD2-15 genotype, enhanced initiation of secondary stem leaves, can be also favourable for recombinant protein production in closed facilities. Recent study demonstrated that axillary stem leaves contributed more than 50% of the recombinant HA H1 yield despite representing only one-third of the total biomass (Goulet et al., 2019).

In this study we showed the potential of the cell cycle regulator gene *At-CycD2* to modulate the host plant for enhanced recombinant protein yield. The At-CycD2-15 transgenic line displayed several features such as improved phenotype and intracellular environment that are relevant to the plant molecular farming application. We used the *Agrobacterium*-mediated transformation technology to modify the *N. benthamiana* genotype. With the availability of the *N. benthamiana* genome assembly (Bombarely et al., 2012) and development of genome editing technologies (TALENs, CRISPR/Cas9) more precise modification of intracellular environment and plant habitus is possible in order to improve *N. benthamiana* as a manufacturing platform for recombinant proteins (Buyel et al., 2021).

## Data Availability Statement

The original contributions presented in the study are included in the article/Supplementary Material, further inquiries can be directed to the corresponding author.

## Author Contributions

LK designed the study, analysed the results, and wrote the manuscript. SR performed statistical analysis of data and created figures. All authors contributed to the article and approved the submitted version.

## Funding

This research was financed in frame of the self-funded JKI project by the German Federal Ministry of Food and Agriculture (BMEL).

## Conflict of Interest

The authors declare that the research was conducted in the absence of any commercial or financial relationships that could be construed as a potential conflict of interest.

## Publisher’s Note

All claims expressed in this article are solely those of the authors and do not necessarily represent those of their affiliated organizations, or those of the publisher, the editors and the reviewers. Any product that may be evaluated in this article, or claim that may be made by its manufacturer, is not guaranteed or endorsed by the publisher.

## Footnotes

^1^ http://rsb.info.nih.gov/ij/
