# Supplementary material for: At-CycD2 Enhances Accumulation of Above-Ground Biomass and Recombinant Proteins in Transgenic Nicotiana benthamiana Plants
Source: Front Plant Sci. 2021 Sep 10;12:712438. doi: 10.3389/fpls.2021.712438 (PMC8460762; doi:10.3389/fpls.2021.712438)
Supplement: Supplementary file 1 [file Data_Sheet_1.pdf]

SUPPLEMENTARY MATERIAL

FIGURES

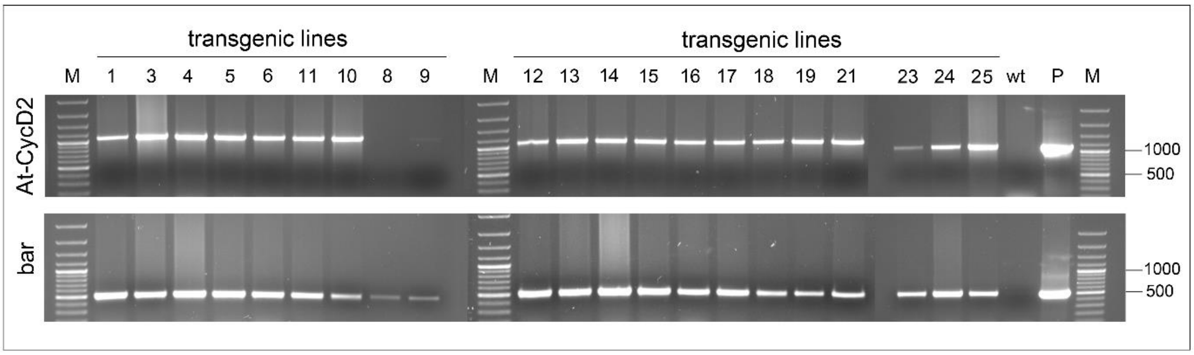

Figure S1. Verification of At-CycD2 transgenic plants by PCR analysis. Genomic DNA from PPT resistant T_0 plants (1, 3, 4, 5, 6, 8, 9, 10, 11, 12, 13, 14, 15, 16, 17, 18, 19, 21, 23, 24 and 25) was subjected to PCR analysis using the At-CycD2-forw/At-CycD2-rev and Bar-forw/Bar-rev primers specific to the *At-CycD2* and *bar* genes, respectively. The At-CycD2 primers amplify a 1070 bp fragment of the corresponding gene, whereas the *bar* primers produce a 503 bp fragment. DNAs of non-transgenic *N. benthamiana* plant (wt) and plasmid pLH-*35S-At-CycD2* (P) were included as negative and positive controls, respectively. GeneRuler 100 bp Plus DNA marker (Thermo Scientific, Waltham, USA; M).

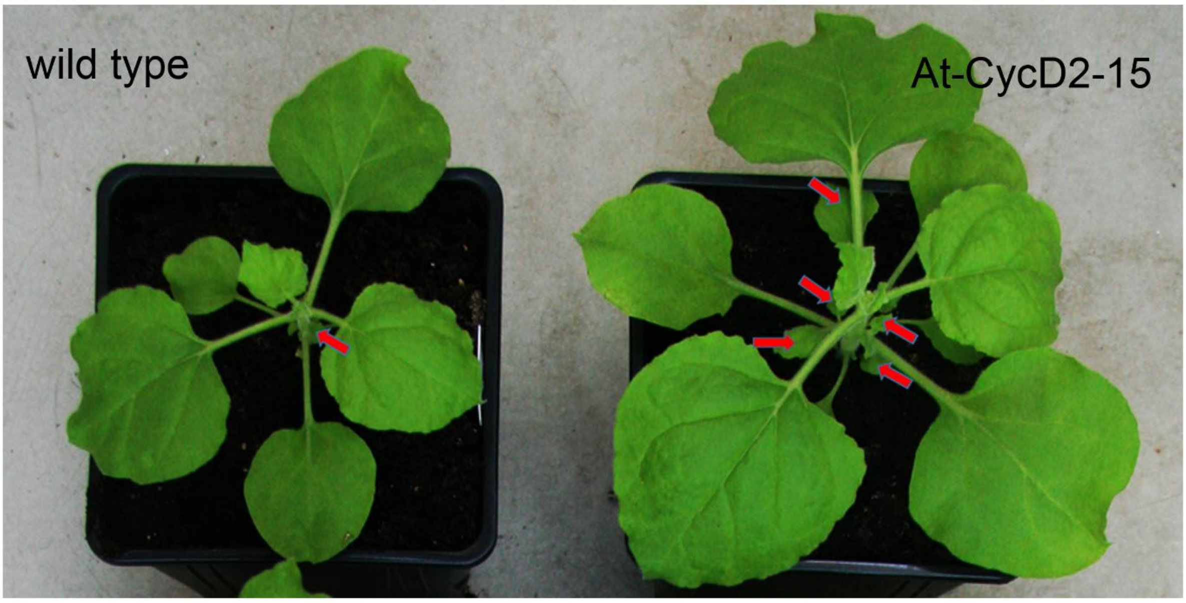

Figure S2. Secondary stem leaf initiation of the At-CycD2 line. The soil-grown plants were photographed at 21 days after planting. Arrows show secondary stem leaves.

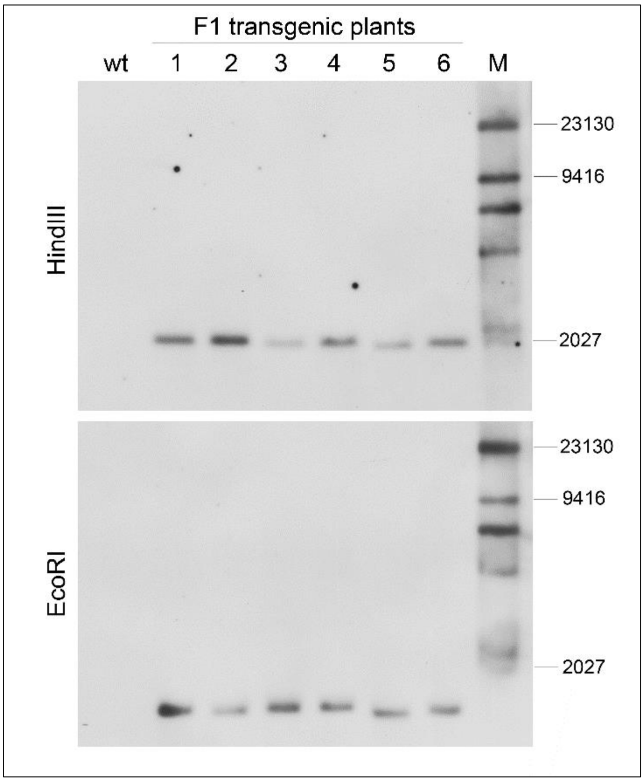

Figure S3. Southern blot analysis of the At-CycD2-15 transgenic line. Genomic DNA from non-transgenic and T_1 progeny plants of the At-CycD2-15 transgenic line was cleaved with *Hind*III (*Eco*RI) and hybridized with the DIG-labelled *bar* probe. Lanes are as follows: wild type *N. benthamiana* (wt), T_1 transgenic plants (1-6), DIG-labelled molecular marker (M).

TABLES

Table S1. Primers used in this study. Primers used for cloning (C), transgene verification (TV); expression analysis (EA) and development of probe for Southern blot analysis (SB) are presented. Restriction sites used for cloning are underlined.

Primer	Sequence (5’-3’)	Application
**Bar-forw**	ATGCCGGCGGTCTGCACCATCGTC	SB
**Bar-rev**	CGCGTGATCTCAGATCTCGGTGAC	SB
**PacI-TM43-E10-forw**	GTCT<u>TTAATTAA</u>ATGGCCGAAGTGCAGCTGGT	C
**TM43-E10-EcoRV-rev**	CGGC<u>GATATC</u>TTAATGATGATGGTGATGAT	C
**At-CycD2-forw**	ACCCCATGGCTGAGAATCTTGCTTGT	TV
**At-CycD2-rev**	CGGTCTAGATCATTGTTTTCTCCTCC	TV, EA
**Bar-forw**	ATGCCGGCGGTCTGCACCATCGTC	TV
**Bar-rev**	CGCGTGATCTCAGATCTCGGTGAC	TV
**GAPDH-238-forw**	GGAGGAGGGAACAACAAGAGG	EA
**GAPDH-238-rev**	AGATGCCGTCAGTGCCGA	EA
**At-CycD2-349-forw**	AGGCCTTCTGAGATAGCTGCAGCTGC	EA

Table S2. Segregation test of T_1 progeny of transgenic *N. benthamiana* lines carrying the *At-CycD2* gene. T_1 seeds were germinated on selective MS medium supplemented with 5 mg/L PPT. Number of PPT resistant (PPT^R) and PPT sensitive (PPT^S) seedlings was calculated at two weeks after sowing.

Line	Number of plants			Ratio
	Total	PPT^R	PPT^S	PPT^R/PPT^S
**At-CycD2-1**	222	165	57	2.9:1
**At-CycD2-3**	100	77	23	3.3:1
**At-CycD2-4**	104	88	16	5.5:1
**At-CycD2-5**	202	177	25	7:1
**At-CycD2-6**	510	395	115	3.4:1
**At-CycD2-10**	167	142	25	5.7:1
**At-CycD2-11**	264	194	70	2.8:1
**At-CycD2-12**	197	163	34	4.8:1
**At-CycD2-13**	69	66	3	22:1
**At-CycD2-14**	409	385	24	16:1
**At-CycD2-15**	623	474	149	3:1
**At-CycD2-16**	211	190	21	9:1
**At-CycD2-17**	247	223	9	24:1
**At-CycD2-18**	156	127	29	4.4:1
**At-CycD2-19**	174	163	21	7.8:1
**At-CycD2-21**	230	176	54	3.3:1
**At-CycD2-24**	199	184	15	12:1
**At-CycD2-25**	430	393	37	11:1

Table S3. Primary data for phenotypic evaluation of transgenic *N. benthamiana* lines expressing the *At-CycD2* gene. Non-transgenic and T_1 progeny of At-CycD2-6, At-CycD2-11, At-CycD2-12, At-CycD2-14, At-CycD2-15, At-CycD2-16, At-CycD2-17, At-CycD2-18, At-CycD2-21, At-CycD2-24 and At-CycD2-25 transgenic plants were grown in greenhouse for 4 weeks and assayed for the plant height, number of leaves and above ground biomass characteristics. The primary data for three repeats are presented (n=45). Each replication included 15 plants per transgenic line or wild type non-transformed control.

At-CycD2-6 vs. wt

genotype	nr.	repeat	plant height, cm	number of leaves	leaf biomass, g	stem biomass, g
At-CycD2-6	1	1	10.8	22	11.8	5.43
At-CycD2-6	2	1	11	15	11.5	5.33
At-CycD2-6	3	1	11.3	15	11.4	5.01
At-CycD2-6	4	1	13.3	20	12.3	6
At-CycD2-6	5	1	12.8	18	11.8	5.5
At-CycD2-6	6	1	11.2	17	11.1	4.83
At-CycD2-6	7	1	15.5	26	16.9	8.97
At-CycD2-6	8	1	14.3	22	13.5	6.73
At-CycD2-6	9	1	13	24	15.4	7.34
At-CycD2-6	10	1	15.3	26	20.2	9.83
At-CycD2-6	11	1	12.2	25	15.6	7.05
At-CycD2-6	12	1	14.3	22	13.7	6.79
At-CycD2-6	13	1	11.8	24	14	6.2
At-CycD2-6	14	1	11.7	23	13.9	6.46
At-CycD2-6	15	1	11.8	20	12.9	5.81
At-CycD2-6	16	2	14.5	24	17.2	7.42
At-CycD2-6	17	2	16	23	17.9	8.45
At-CycD2-6	18	2	12.6	29	22.1	9.79
At-CycD2-6	19	2	14.7	27	20.4	9.53
At-CycD2-6	20	2	13.8	17	19.1	4.6
At-CycD2-6	21	2	16.5	15	11.5	6.12
At-CycD2-6	22	2	11	21	13.8	5.42
At-CycD2-6	23	2	12.6	21	14.1	6.2
At-CycD2-6	24	2	15.2	17	11.7	6
At-CycD2-6	25	2	14.5	22	16.2	7.41
At-CycD2-6	26	2	16.5	20	16.6	8.52
At-CycD2-6	27	2	15.2	30	22.3	10.2
At-CycD2-6	28	2	14	27	19.2	8.11
At-CycD2-6	29	2	15.2	23	17.4	7.69
At-CycD2-6	30	2	14.8	32	23.2	10.8
At-CycD2-6	31	3	10	23	13.9	5.49
At-CycD2-6	32	3	11.7	20	12	5.63
At-CycD2-6	33	3	12.4	23	11.8	5.59
At-CycD2-6	34	3	9.7	27	14.3	5.74
At-CycD2-6	35	3	11.2	22	13.6	5.8
At-CycD2-6	36	3	11	22	13.6	5.92
At-CycD2-6	37	3	10.2	22	12.1	5.38
At-CycD2-6	38	3	11.8	22	11.6	5.23
At-CycD2-6	39	3	10.9	28	16.1	6.74
At-CycD2-6	40	3	9.3	19	15	5.18
At-CycD2-6	41	3	11	22	14.7	4.88
At-CycD2-6	42	3	10.8	20	15	4.98
At-CycD2-6	43	3	10.9	20	14.7	5.3
At-CycD2-6	44	3	9.3	19	19.4	5.79
At-CycD2-6	45	3	10.5	20	11.9	5.92

genotype	nr.	repeat	plant height, cm	number of leaves	leaf biomass, g	stem biomass, g
wt	1	1	12.7	18	10.1	5.93
wt	2	1	12.2	22	10.6	5.63
wt	3	1	13.0	25	11.5	6.63
wt	4	1	12.0	21	9.3	5.26
wt	5	1	12.5	20	10.8	5.3
wt	6	1	12.2	22	10.9	5.61
wt	7	1	13.8	21	10.5	5.75
wt	8	1	13.2	24	11.6	6.2
wt	9	1	13.5	20	10.5	5.2
wt	10	1	13.2	21	10.8	5.88
wt	11	1	12.3	23	9.84	5.6
wt	12	1	11	19	8.44	4.19
wt	13	1	13	26	11.3	5.95
wt	14	1	13.6	23	10.4	5.65
wt	15	1	7.8	11	3.89	1.74
wt	16	2	16.2	24	13	6.58
wt	17	2	11.8	23	10.7	6.32
wt	18	2	16.0	22	11.3	5.74
wt	19	2	12.5	19	9.35	4.29
wt	20	2	17.0	22	10.6	5.76
wt	21	2	16.6	24	11.1	4.66
wt	22	2	18	25	15.8	5.18
wt	23	2	16.2	19	10.5	5.07
wt	24	2	13	18	11.6	4.86
wt	25	2	17.5	22	13.7	6.72
wt	26	2	18.3	27	12	4.66
wt	27	2	18.2	19	10.9	6.41
wt	28	2	15	25	14.2	6.62
wt	29	2	15.5	19	11.1	5.23
wt	30	2	14.3	23	12.8	5.5
wt	31	3	14.5	20	12.3	4.18
wt	32	3	11.3	20	12.6	5.81
wt	33	3	12.0	26	12.4	5.95
wt	34	3	12.7	19	14.8	5.67
wt	35	3	12.2	19	14.4	4.41
wt	36	3	12.0	24	11	4.78
wt	37	3	13.2	18	7.73	4.19
wt	38	3	13.2	25	12.3	5.81
wt	39	3	12.6	19	10.2	3.45
wt	40	3	11.7	25	13.8	4.03
wt	41	3	11.2	26	11.5	4.83
wt	42	3	12.2	20	12.7	5.61
wt	43	3	11.2	26	10.5	4.62
wt	44	3	11.6	25	10.6	4.79
wt	45	3	11.8	19	11.1	4.72

At-CycD2-11 vs. wt

genotype	nr.	repeat	plant height, cm	number of leaves	leaf biomass, g	stem biomass, g
At-CycD2-11	1	1	12.2	20	9.59	4.8
At-CycD2-11	2	1	11.3	17	8.43	4.1
At-CycD2-11	3	1	10.3	21	9.8	4.87
At-CycD2-11	4	1	12	15	10.3	5.14
At-CycD2-11	5	1	13.3	20	9.94	5.62
At-CycD2-11	6	1	14	18	12.3	6.33
At-CycD2-11	7	1	14.2	18	10.9	6
At-CycD2-11	8	1	12	17	10.2	5.25
At-CycD2-11	9	1	13.2	22	12.1	6.39
At-CycD2-11	10	1	13	19	9.04	4.97
At-CycD2-11	11	1	12.5	21	10.3	5.45
At-CycD2-11	12	1	13.8	15	10.9	5.84
At-CycD2-11	13	1	13.3	18	11.2	6.13
At-CycD2-11	14	1	13.2	20	10.8	5.45
At-CycD2-11	15	1	11.2	19	8.41	4.02
At-CycD2-11	16	2	18.5	29	18.6	10.5
At-CycD2-11	17	2	20	32	19.2	11.3
At-CycD2-11	18	2	19.4	27	17.9	10.4
At-CycD2-11	19	2	21	28	18.6	11.7
At-CycD2-11	20	2	16.2	18	8.7	5.13
At-CycD2-11	21	2	21	24	15.7	9.55
At-CycD2-11	22	2	17.2	28	16.9	9.7
At-CycD2-11	23	2	22	32	18.9	12.3
At-CycD2-11	24	2	21.5	35	21.8	13.5
At-CycD2-11	25	2	24.2	31	18.9	12.3
At-CycD2-11	26	2	21.7	28	16.4	10.2
At-CycD2-11	27	2	22	25	17.3	10.1
At-CycD2-11	28	2	21.7	30	20.1	12.6
At-CycD2-11	29	2	20.8	27	16.9	10.6
At-CycD2-11	30	2	17	27	17	10.2
At-CycD2-11	31	3	26.1	33	18.93	10.92
At-CycD2-11	32	3	28.2	36	21.12	13.05
At-CycD2-11	33	3	24.1	33	20.60	11.07
At-CycD2-11	34	3	27.3	38	25.03	14.66
At-CycD2-11	35	3	25.8	28	14.92	9.39
At-CycD2-11	36	3	28.2	32	19.18	11.29
At-CycD2-11	37	3	27.1	40	20.66	11.40
At-CycD2-11	38	3	30.0	42	26.97	15.90
At-CycD2-11	39	3	26.9	28	19.23	11.18
At-CycD2-11	40	3	29.8	36	22.26	13.45
At-CycD2-11	41	3	25.1	29	19.22	10.92
At-CycD2-11	42	3	22.4	21	14.12	8.29
At-CycD2-11	43	3	28.1	35	22.76	13.17
At-CycD2-11	44	3	24.2	23	14.90	9.11
At-CycD2-11	45	3	26.5	25	16.80	10.32

genotype	nr.	repeat	plant height, cm	number of leaves	leaf biomass, g	stem biomass, g
wt	1	1	12.7	18	10.1	5.93
wt	2	1	12.2	22	10.6	5.63
wt	3	1	13.0	25	11.5	6.63
wt	4	1	12.0	21	9.3	5.26
wt	5	1	12.5	20	10.8	5.3
wt	6	1	12.2	22	10.9	5.61
wt	7	1	13.8	21	10.5	5.75
wt	8	1	13.2	24	11.6	6.2
wt	9	1	13.5	20	10.5	5.2
wt	10	1	13.2	21	10.8	5.88
wt	11	1	12.3	23	9.84	5.6
wt	12	1	11	19	8.44	4.19
wt	13	1	13	26	11.3	5.95
wt	14	1	13.6	23	10.4	5.65
wt	15	1	7.8	11	3.89	1.74
wt	16	2	16.3	30	13.4	7.98
wt	17	2	20.6	30	17.9	11.1
wt	18	2	25.5	41	23.5	15.2
wt	19	2	22.7	39	23.3	13.5
wt	20	2	20.7	35	18.3	10.1
wt	21	2	22.0	36	21.6	13.4
wt	22	2	22.3	31	15.8	10.1
wt	23	2	21	33	19	10.4
wt	24	2	17.2	28	12.7	6.7
wt	25	2	17.3	30	16.8	9.12
wt	26	2	21	28	15.3	8.52
wt	27	2	19.6	34	18.8	11
wt	28	2	17.1	28	13.6	7.44
wt	29	2	17.6	32	14.9	7.69
wt	30	2	19.8	37	17.2	9.84
wt	31	3	22.0	37	15.36	8.31
wt	32	3	27.3	39	18.42	8.66
wt	33	3	23.2	41	20.87	10.93
wt	34	3	25.4	36	18.74	10.24
wt	35	3	26.4	32	15.93	9.53
wt	36	3	27.7	32	16.13	9.66
wt	37	3	25.2	34	14.89	8.56
wt	38	3	23.6	30	13.93	7.58
wt	39	3	26.2	40	17.90	10.46
wt	40	3	24.3	32	15.65	8.50
wt	41	3	26.5	35	15.38	8.79
wt	42	3	27	36	16.28	9.27
wt	43	3	28.1	40	20.93	10.64
wt	44	3	20.1	36	14.84	7.66
wt	45	3	26.4	37	13.51	9.00

At-CycD2-12 vs. wt

genotype	nr.	repeat	plant height, cm	number of leaves	leaf biomass, g	stem biomass, g
At-CycD2-12	1	1	9.7	17	8.16	3.74
At-CycD2-12	2	1	10.7	15	8.26	4.02
At-CycD2-12	3	1	7.1	8	4.34	1.84
At-CycD2-12	4	1	10.6	12	9.12	3.93
At-CycD2-12	5	1	11.4	16	8.11	3.76
At-CycD2-12	6	1	7	11	5.31	2.23
At-CycD2-12	7	1	10.8	13	4.46	2.32
At-CycD2-12	8	1	12.9	15	9.67	4.43
At-CycD2-12	9	1	10.3	15	8.09	3.8
At-CycD2-12	10	1	8.7	12	8.15	3.12
At-CycD2-12	11	1	12.2	12	4.73	2.42
At-CycD2-12	12	1	9.8	15	6.55	2.76
At-CycD2-12	13	1	10.7	11	8.92	3.79
At-CycD2-12	14	1	9	16	5.36	2.37
At-CycD2-12	15	1	6.7	13	4.15	1.78
At-CycD2-12	16	2	24.4	47	32.9	21.4
At-CycD2-12	17	2	25.6	46	28.5	19.1
At-CycD2-12	18	2	27.2	43	29	19.2
At-CycD2-12	19	2	27.1	40	25.7	16.9
At-CycD2-12	20	2	26.3	36	26.8	16.6
At-CycD2-12	21	2	29.5	38	26.8	18.8
At-CycD2-12	22	2	28.1	43	28	21.6
At-CycD2-12	23	2	24.4	43	19.4	11.5
At-CycD2-12	24	2	28.9	40	27.4	19.4
At-CycD2-12	25	2	27.8	37	21.8	13.5
At-CycD2-12	26	2	30	38	23.8	14.3
At-CycD2-12	27	2	30.1	49	23.2	17.8
At-CycD2-12	28	2	24.4	41	25.2	15.4
At-CycD2-12	29	2	31.1	44	30.7	21.7
At-CycD2-12	30	2	27.6	31	26.2	17
At-CycD2-12	31	3	30.0	44	28.2	16.13
At-CycD2-12	32	3	23.3	40	20.2	12.52
At-CycD2-12	33	3	26.2	42	25	14.14
At-CycD2-12	34	3	19.2	30	16.5	7.99
At-CycD2-12	35	3	29.2	40	23	12.80
At-CycD2-12	36	3	25.9	33	20.3	11.07
At-CycD2-12	37	3	30.9	35	25.7	14.42
At-CycD2-12	38	3	30.8	33	21.8	13.34
At-CycD2-12	39	3	30.2	35	21.6	13.14
At-CycD2-12	40	3	22.9	29	18.4	10.46
At-CycD2-12	41	3	30.4	42	26.9	15.98
At-CycD2-12	42	3	27.9	26	18	11.51
At-CycD2-12	43	3	21.4	31	16.9	9.07
At-CycD2-12	44	3	27.4	37	24.3	13.33
At-CycD2-12	45	3	26.3	32	17	10.18

genotype	nr.	repeat	plant height, cm	number of leaves	leaf biomass, g	stem biomass, g
wt	1	1	11.5	25	8.6	4.12
wt	2	1	10.4	21	8.2	3.75
wt	3	1	12.4	27	10.9	5.29
wt	4	1	9.3	17	4.84	2.06
wt	5	1	9.8	14	4.8	2.14
wt	6	1	11.7	21	7.99	3.82
wt	7	1	10.6	22	7.39	3.22
wt	8	1	8.2	11	3.39	1.79
wt	9	1	10	18	6.18	2.81
wt	10	1	10.7	20	8.71	4.1
wt	11	1	11.4	24	9.06	4.42
wt	12	1	11.8	26	11.7	5.53
wt	13	1	12.5	26	11.5	5.36
wt	14	1	12.7	29	12.4	6.09
wt	15	1	11.7	23	11.2	5.41
wt	16	2	24.4	40	26.7	16.2
wt	17	2	26.4	43	20.7	13.6
wt	18	2	26.6	53	22.9	15.8
wt	19	2	27.6	31	15.7	10.3
wt	20	2	27.4	49	22.4	15.3
wt	21	2	28.7	54	29.5	21.3
wt	22	2	28.4	38	22.8	14.7
wt	23	2	27.5	40	22.4	15.4
wt	24	2	29	38	24.5	17.6
wt	25	2	28.4	42	23.9	17
wt	26	2	28.4	44	23	15.9
wt	27	2	26.2	40	26.1	16
wt	28	2	26.3	44	22	12.6
wt	29	2	27.6	38	21.2	14.4
wt	30	2	22.2	37	18.2	12
wt	31	3	22.0	37	15.36	8.31
wt	32	3	27.3	39	18.42	8.66
wt	33	3	23.2	41	20.87	10.93
wt	34	3	25.4	36	18.74	10.24
wt	35	3	26.4	32	15.93	9.53
wt	36	3	27.7	32	16.13	9.66
wt	37	3	25.2	34	14.89	8.56
wt	38	3	23.6	30	13.93	7.58
wt	39	3	26.2	40	17.90	10.46
wt	40	3	24.3	32	15.65	8.50
wt	41	3	26.5	35	15.38	8.79
wt	42	3	27	36	16.28	9.27
wt	43	3	28.1	40	20.93	10.64
wt	44	3	20.1	36	14.84	7.66
wt	45	3	26.4	37	13.51	9.00

At-CycD2-14 vs. wt

genotype	nr.	repeat	plant height, cm	number of leaves	leaf biomass, g	stem biomass, g
At-CycD2-14	1	1	10.8	20	15	6.64
At-CycD2-14	2	1	12.5	21	18	8.96
At-CycD2-14	3	1	10.5	20	14	6.48
At-CycD2-14	4	1	10.5	19	13.9	6.13
At-CycD2-14	5	1	9.7	18	13.9	5.52
At-CycD2-14	6	1	12	23	17.6	6.96
At-CycD2-14	7	1	11.7	26	17.7	8.32
At-CycD2-14	8	1	10	20	16.1	6.56
At-CycD2-14	9	1	11.2	20	15.4	6.12
At-CycD2-14	10	1	10.2	22	17.5	7.2
At-CycD2-14	11	1	10.5	21	15.7	7.18
At-CycD2-14	12	1	10.5	27	19.8	8.5
At-CycD2-14	13	1	11.4	31	19.4	8.28
At-CycD2-14	14	1	10.4	23	17.3	6.75
At-CycD2-14	15	1	10.8	24	19.4	7.4
At-CycD2-14	16	2	8.7	22	16.3	5.14
At-CycD2-14	17	2	9.8	23	17.2	5.8
At-CycD2-14	18	2	8.5	22	14.9	4.84
At-CycD2-14	19	2	8	19	16.8	4.69
At-CycD2-14	20	2	7.8	17	8.2	3.09
At-CycD2-14	21	2	9.6	18	11.8	3.75
At-CycD2-14	22	2	7.5	22	13.3	4.53
At-CycD2-14	23	2	8.3	21	13.9	4.95
At-CycD2-14	24	2	9.5	20	15.1	4.99
At-CycD2-14	25	2	8.8	22	16.2	5.23
At-CycD2-14	26	2	7	19	15.3	4.15
At-CycD2-14	27	2	9.2	23	17.2	5.22
At-CycD2-14	28	2	8.5	27	16.3	5.25
At-CycD2-14	29	2	8.5	21	13.1	4.55
At-CycD2-14	30	2	9.4	25	19.1	6.29
At-CycD2-14	31	3	13.3	28	14.9	6.72
At-CycD2-14	32	3	10.9	16	19.1	6.71
At-CycD2-14	33	3	12	17	9.33	4.45
At-CycD2-14	34	3	17.5	25	17	9.46
At-CycD2-14	35	3	15.3	16	11.5	5.28
At-CycD2-14	36	3	19	19	11.5	7.3
At-CycD2-14	37	3	16.4	26	14.8	7.67
At-CycD2-14	38	3	16	24	14.8	7.41
At-CycD2-14	39	3	10.5	25	17.4	7.33
At-CycD2-14	40	3	15.5	22	12.9	6.45
At-CycD2-14	41	3	14	18	10.3	4.75
At-CycD2-14	42	3	13.8	17	19.2	4.63
At-CycD2-14	43	3	14.8	14	9.65	5.65
At-CycD2-14	44	3	14.3	13	17.3	4.73
At-CycD2-14	45	3	12.6	16	18	3.9

genotype	nr.	repeat	plant height, cm	number of leaves	leaf biomass, g	stem biomass, g
wt	1	1	10.8	19	12.4	5.7
wt	2	1	11.6	18	12.9	5.46
wt	3	1	10.0	21	12.1	5.15
wt	4	1	10.6	19	10.8	5.02
wt	5	1	8.3	20	12.1	4.52
wt	6	1	10.5	18	9.78	4.2
wt	7	1	9	17	8.37	3.57
wt	8	1	9.8	19	10.2	4.16
wt	9	1	10.9	20	11.3	4.84
wt	10	1	11.5	24	12.9	6
wt	11	1	11	17	9.73	4.44
wt	12	1	9.2	17	7.25	2.92
wt	13	1	11	15	8.31	3.86
wt	14	1	12.2	25	10.2	4.8
wt	15	1	11.3	18	10.3	4.56
wt	16	2	10.5	19	8.11	3.13
wt	17	2	14.8	23	14.6	6.94
wt	18	2	13.2	26	17.2	7.89
wt	19	2	11.8	17	9.37	4.25
wt	20	2	11.5	18	14.1	5.65
wt	21	2	13.2	18	11.8	5.13
wt	22	2	10.5	14	9.38	3.8
wt	23	2	13.5	23	14.6	6.77
wt	24	2	14.5	24	11.5	5.69
wt	25	2	13.7	26	16.1	6.84
wt	26	2	11.4	17	10.3	3.97
wt	27	2	12.1	20	13.4	5.33
wt	28	2	10.8	16	9.36	3.84
wt	29	2	11.7	20	10.5	4.6
wt	30	2	10	14	7.56	3.14
wt	31	3	16.2	24	13	6.58
wt	32	3	11.8	23	10.7	6.32
wt	33	3	16.0	22	11.3	5.74
wt	34	3	12.5	19	9.35	4.29
wt	35	3	17.0	22	10.6	5.76
wt	36	3	16.6	24	11.1	4.66
wt	37	3	18	25	15.8	5.18
wt	38	3	16.2	19	10.5	5.07
wt	39	3	13	18	11.6	4.86
wt	40	3	17.5	22	13.7	6.72
wt	41	3	18.3	27	12	4.66
wt	42	3	18.2	19	10.9	6.41
wt	43	3	15	25	14.2	6.62
wt	44	3	15.5	19	11.1	5.23
wt	45	3	14.3	23	12.8	5.5

At-CycD2-15 vs. wt

genotype	nr.	repeat	plant height, cm	number of leaves	leaf biomass, g	stem biomass, g
At-CycD2-15	1	1	13.8	28	15.4	8.59
At-CycD2-15	2	1	12.6	26	15.6	8.1
At-CycD2-15	3	1	11.8	22	11.1	5.89
At-CycD2-15	4	1	13.7	29	13.2	7.25
At-CycD2-15	5	1	11.2	25	10.7	5.62
At-CycD2-15	6	1	12	25	11.5	6.37
At-CycD2-15	7	1	13.7	25	12.4	7.2
At-CycD2-15	8	1	12	23	13.2	7.07
At-CycD2-15	9	1	12.2	29	12.1	6.97
At-CycD2-15	10	1	13.6	23	12.5	7.11
At-CycD2-15	11	1	13.5	29	14.1	8.33
At-CycD2-15	12	1	14.5	28	16.7	9.76
At-CycD2-15	13	1	14.5	30	15	8.73
At-CycD2-15	14	1	13.3	24	15	7.99
At-CycD2-15	15	1	13	24	13.5	7.18
At-CycD2-15	16	2	11.3	31	21.2	8.37
At-CycD2-15	17	2	9.5	22	19.4	6.84
At-CycD2-15	18	2	7.8	22	16	5.39
At-CycD2-15	19	2	10.3	27	20.7	7.68
At-CycD2-15	20	2	9.2	31	17	7.07
At-CycD2-15	21	2	9	29	16.6	6.65
At-CycD2-15	22	2	10.5	29	21	8.34
At-CycD2-15	23	2	11.2	31	23	9.85
At-CycD2-15	24	2	9	25	16.8	6.49
At-CycD2-15	25	2	9.2	23	16.9	5.83
At-CycD2-15	26	2	10.2	31	22.2	8.63
At-CycD2-15	27	2	9.4	28	17	6.78
At-CycD2-15	28	2	10.4	28	21.7	8.41
At-CycD2-15	29	2	9.4	30	18.6	7.36
At-CycD2-15	30	2	10.6	31	23.2	8.93
At-CycD2-15	31	3	12.3	21	12.9	6.56
At-CycD2-15	32	3	13	24	16.1	7.38
At-CycD2-15	33	3	11.8	20	13.1	6.75
At-CycD2-15	34	3	14.6	18	14.1	6.29
At-CycD2-15	35	3	14	31	22.4	9.76
At-CycD2-15	36	3	13.4	28	20.8	9.17
At-CycD2-15	37	3	13.4	24	10.3	6.51
At-CycD2-15	38	3	13.6	22	17.4	8.61
At-CycD2-15	39	3	11.5	20	12.9	6.98
At-CycD2-15	40	3	12	25	12	6.42
At-CycD2-15	41	3	16.3	20	15.4	8.61
At-CycD2-15	42	3	15.4	22	14.5	6.8
At-CycD2-15	43	3	12	21	16	7.08
At-CycD2-15	44	3	12.5	19	10.2	4.46
At-CycD2-15	45	3	17.5	30	23.7	11.9

genotype	nr.	repeat	plant height, cm	number of leaves	leaf biomass, g	stem biomass, g
wt	1	1	12.7	18	10.1	5.93
wt	2	1	12.2	22	10.6	5.63
wt	3	1	13.0	25	11.5	6.63
wt	4	1	12.0	21	9.3	5.26
wt	5	1	12.5	20	10.8	5.3
wt	6	1	12.2	22	10.9	5.61
wt	7	1	13.8	21	10.5	5.75
wt	8	1	13.2	24	11.6	6.2
wt	9	1	13.5	20	10.5	5.2
wt	10	1	13.2	21	10.8	5.88
wt	11	1	12.3	23	9.84	5.6
wt	12	1	11	19	8.44	4.19
wt	13	1	13	26	11.3	5.95
wt	14	1	13.6	23	10.4	5.65
wt	15	1	7.8	11	3.89	1.74
wt	16	2	10.5	19	8.11	3.13
wt	17	2	14.8	23	14.6	6.94
wt	18	2	13.2	26	17.2	7.89
wt	19	2	11.8	17	9.37	4.25
wt	20	2	11.5	18	14.1	5.65
wt	21	2	13.2	18	11.8	5.13
wt	22	2	10.5	14	9.38	3.8
wt	23	2	13.5	23	14.6	6.77
wt	24	2	14.5	24	11.5	5.69
wt	25	2	13.7	26	16.1	6.84
wt	26	2	11.4	17	10.3	3.97
wt	27	2	12.1	20	13.4	5.33
wt	28	2	10.8	16	9.36	3.84
wt	29	2	11.7	20	10.5	4.6
wt	30	2	10	14	7.56	3.14
wt	31	3	16.2	24	13	6.58
wt	32	3	11.8	23	10.7	6.32
wt	33	3	16.0	22	11.3	5.74
wt	34	3	12.5	19	9.35	4.29
wt	35	3	17.0	22	10.6	5.76
wt	36	3	16.6	24	11.1	4.66
wt	37	3	18	25	15.8	5.18
wt	38	3	16.2	19	10.5	5.07
wt	39	3	13	18	11.6	4.86
wt	40	3	17.5	22	13.7	6.72
wt	41	3	18.3	27	12	4.66
wt	42	3	18.2	19	10.9	6.41
wt	43	3	15	25	14.2	6.62
wt	44	3	15.5	19	11.1	5.23
wt	45	3	14.3	23	12.8	5.5

At-CycD2-16 vs. wt

genotype	nr.	repeat	plant height, cm	number of leaves	leaf biomass, g	stem biomass, g
At-CycD2-16	1	1	9.5	25	7.74	3.37
At-CycD2-16	2	1	13.4	25	9.27	5.02
At-CycD2-16	3	1	14.1	21	13.3	6.28
At-CycD2-16	4	1	14.5	23	16.5	6.88
At-CycD2-16	5	1	15	36	13.3	6.22
At-CycD2-16	6	1	14.2	27	13.3	6.09
At-CycD2-16	7	1	13	22	14.2	5.72
At-CycD2-16	8	1	12.4	16	12.1	5.37
At-CycD2-16	9	1	11.5	20	11.7	4.3
At-CycD2-16	10	1	11.8	16	11.7	5.2
At-CycD2-16	11	1	12.3	25	9.77	4.56
At-CycD2-16	12	1	12.2	26	13.3	5.98
At-CycD2-16	13	1	11	21	9.3	3.97
At-CycD2-16	14	1	13.5	20	6.68	3.66
At-CycD2-16	15	1	11.8	20	8.39	3.43
At-CycD2-16	16	2	28	36	24.1	12.6
At-CycD2-16	17	2	27	19	16.6	10.9
At-CycD2-16	18	2	19.2	19	13.8	7.93
At-CycD2-16	19	2	24.9	18	17.2	9.47
At-CycD2-16	20	2	25.7	23	15.9	8.67
At-CycD2-16	21	2	29.3	56	32.4	19.7
At-CycD2-16	22	2	25.1	18	15.2	9.58
At-CycD2-16	23	2	27.6	17	27.2	14.5
At-CycD2-16	24	2	26.1	21	31.4	15.3
At-CycD2-16	25	2	29.3	37	18.6	12.4
At-CycD2-16	26	2	24.8	27	25.6	13.6
At-CycD2-16	27	2	23	27	15.5	8.76
At-CycD2-16	28	2	24.5	34	36.4	17.1
At-CycD2-16	29	2	24.3	24	10	5.36
At-CycD2-16	30	2	23.7	27	14.4	8.5
At-CycD2-16	31	3	26.1	28	25.95	12.12
At-CycD2-16	32	3	22.2	24	16.65	8.24
At-CycD2-16	33	3	24.5	37	15.59	7.29
At-CycD2-16	34	3	22.7	27	13.48	6.59
At-CycD2-16	35	3	25.6	26	20.19	10.19
At-CycD2-16	36	3	25.0	30	26.48	11.50
At-CycD2-16	37	3	25.2	28	23.85	9.42
At-CycD2-16	38	3	24.2	21	9.00	5.05
At-CycD2-16	39	3	22.4	23	17.58	8.71
At-CycD2-16	40	3	24.2	32	14.39	7.07
At-CycD2-16	41	3	20.2	18	13.00	5.97
At-CycD2-16	42	3	20.6	30	13.37	6.64
At-CycD2-16	43	3	21.0	45	14.87	6.46
At-CycD2-16	44	3	25.4	33	27.65	14.42
At-CycD2-16	45	3	22.9	22	10.72	5.20

genotype	nr.	repeat	plant height, cm	number of leaves	leaf biomass, g	stem biomass, g
wt	1	1	11.5	25	8.6	4.12
wt	2	1	10.4	21	8.2	3.75
wt	3	1	12.4	27	10.9	5.29
wt	4	1	9.3	17	4.84	2.06
wt	5	1	9.8	14	4.8	2.14
wt	6	1	11.7	21	7.99	3.82
wt	7	1	10.6	22	7.39	3.22
wt	8	1	8.2	11	3.39	1.79
wt	9	1	10	18	6.18	2.81
wt	10	1	10.7	20	8.71	4.1
wt	11	1	11.4	24	9.06	4.42
wt	12	1	11.8	26	11.7	5.53
wt	13	1	12.5	26	11.5	5.36
wt	14	1	12.7	29	12.4	6.09
wt	15	1	11.7	23	11.2	5.41
wt	16	2	24.4	40	26.7	16.2
wt	17	2	26.4	43	20.7	13.6
wt	18	2	26.6	53	22.9	15.8
wt	19	2	27.6	31	15.7	10.3
wt	20	2	27.4	49	22.4	15.3
wt	21	2	28.7	54	29.5	21.3
wt	22	2	28.4	38	22.8	14.7
wt	23	2	27.5	40	22.4	15.4
wt	24	2	29	38	24.5	17.6
wt	25	2	28.4	42	23.9	17
wt	26	2	28.4	44	23	15.9
wt	27	2	26.2	40	26.1	16
wt	28	2	26.3	44	22	12.6
wt	29	2	27.6	38	21.2	14.4
wt	30	2	22.2	37	18.2	12
wt	31	3	22.0	37	15.36	8.31
wt	32	3	27.3	39	18.42	8.66
wt	33	3	23.2	41	20.87	10.93
wt	34	3	25.4	36	18.74	10.24
wt	35	3	26.4	32	15.93	9.53
wt	36	3	27.7	32	16.13	9.66
wt	37	3	25.2	34	14.89	8.56
wt	38	3	23.6	30	13.93	7.58
wt	39	3	26.2	40	17.90	10.46
wt	40	3	24.3	32	15.65	8.50
wt	41	3	26.5	35	15.38	8.79
wt	42	3	27	36	16.28	9.27
wt	43	3	28.1	40	20.93	10.64
wt	44	3	20.1	36	14.84	7.66
wt	45	3	26.4	37	13.51	9.00

At-CycD2-17 vs. wt

genotype	nr.	repeat	plant height, cm	number of leaves	leaf biomass, g	stem biomass, g
At-CycD2-17	1	1	10.5	16	6.36	2.94
At-CycD2-17	2	1	12.2	20	10.4	5.1
At-CycD2-17	3	1	11	19	8.2	3.63
At-CycD2-17	4	1	13.6	19	10.2	5.1
At-CycD2-17	5	1	9.6	18	7.05	3.45
At-CycD2-17	6	1	11.8	21	10.8	4.83
At-CycD2-17	7	1	13.2	20	10.2	5.59
At-CycD2-17	8	1	12.7	19	10.1	5.29
At-CycD2-17	9	1	10.5	19	8.43	3.99
At-CycD2-17	10	1	11.5	18	8.25	3.77
At-CycD2-17	11	1	9.5	20	7.93	3.77
At-CycD2-17	12	1	8.5	19	6.75	3.08
At-CycD2-17	13	1	12.8	20	7.85	4.61
At-CycD2-17	14	1	10.8	20	7.53	3.65
At-CycD2-17	15	1	10.8	19	7.17	3.54
At-CycD2-17	16	2	20.2	27	16.7	8.65
At-CycD2-17	17	2	21	32	20.9	12.3
At-CycD2-17	18	2	19.2	27	16.8	9.48
At-CycD2-17	19	2	18.8	27	16.9	9.97
At-CycD2-17	20	2	19	22	12.4	6.97
At-CycD2-17	21	2	20.2	24	15.4	8.95
At-CycD2-17	22	2	20	28	19.3	10.5
At-CycD2-17	23	2	21	23	14.6	8.74
At-CycD2-17	24	2	17.8	20	13.3	7.34
At-CycD2-17	25	2	17	29	16.6	8.75
At-CycD2-17	26	2	17.5	19	9.96	5.39
At-CycD2-17	27	2	20.3	28	17.6	10.5
At-CycD2-17	28	2	20.5	28	16.8	8.01
At-CycD2-17	29	2	16.8	25	13.9	7.52
At-CycD2-17	30	2	18.3	19	9.58	5.08
At-CycD2-17	31	3	21.1	26	15.79	8.80
At-CycD2-17	32	3	24.4	34	20.43	11.68
At-CycD2-17	33	3	23.9	23	12.61	5.80
At-CycD2-17	34	3	26.0	30	18.46	10.60
At-CycD2-17	35	3	22.8	20	13.40	8.10
At-CycD2-17	36	3	23.7	20	12.25	7.83
At-CycD2-17	37	3	28.9	34	20.96	12.49
At-CycD2-17	38	3	26.2	20	15.65	10.17
At-CycD2-17	39	3	25.3	24	16.28	9.23
At-CycD2-17	40	3	25.3	24	15.54	7.91
At-CycD2-17	41	3	21.4	13	7.66	4.44
At-CycD2-17	42	3	25.2	19	13.99	9.21
At-CycD2-17	43	3	26.3	28	16.65	10.13
At-CycD2-17	44	3	21.5	15	7.98	4.30
At-CycD2-17	45	3	23.4	28	14.18	7.99

genotype	nr.	repeat	plant height, cm	number of leaves	leaf biomass, g	stem biomass, g
wt	1	1	12.7	18	10.1	5.93
wt	2	1	12.2	22	10.6	5.63
wt	3	1	13.0	25	11.5	6.63
wt	4	1	12.0	21	9.3	5.26
wt	5	1	12.5	20	10.8	5.3
wt	6	1	12.2	22	10.9	5.61
wt	7	1	13.8	21	10.5	5.75
wt	8	1	13.2	24	11.6	6.2
wt	9	1	13.5	20	10.5	5.2
wt	10	1	13.2	21	10.8	5.88
wt	11	1	12.3	23	9.84	5.6
wt	12	1	11	19	8.44	4.19
wt	13	1	13	26	11.3	5.95
wt	14	1	13.6	23	10.4	5.65
wt	15	1	7.8	11	3.89	1.74
wt	16	2	16.3	30	13.4	7.98
wt	17	2	20.6	30	17.9	11.1
wt	18	2	25.5	41	23.5	15.2
wt	19	2	22.7	39	23.3	13.5
wt	20	2	20.7	35	18.3	10.1
wt	21	2	22.0	36	21.6	13.4
wt	22	2	22.3	31	15.8	10.1
wt	23	2	21	33	19	10.4
wt	24	2	17.2	28	12.7	6.7
wt	25	2	17.3	30	16.8	9.12
wt	26	2	21	28	15.3	8.52
wt	27	2	19.6	34	18.8	11
wt	28	2	17.1	28	13.6	7.44
wt	29	2	17.6	32	14.9	7.69
wt	30	2	19.8	37	17.2	9.84
wt	31	3	28.1	42	24.69	14.72
wt	32	3	28.7	40	23.24	13.80
wt	33	3	29.1	44	26.51	15.16
wt	34	3	28.2	44	24.87	15.24
wt	35	3	32.2	44	26.96	16.69
wt	36	3	25.3	38	18.89	11.44
wt	37	3	24.9	35	19.92	10.58
wt	38	3	26.3	33	21.16	12.11
wt	39	3	28.6	40	23.19	13.92
wt	40	3	28.4	29	16.99	9.74
wt	41	3	28.3	36	20.90	12.88
wt	42	3	26.9	37	18.51	10.26
wt	43	3	28.6	44	23.34	13.78
wt	44	3	27.7	44	25.88	13.95
wt	45	3	27.6	35	19.54	10.89

At-CycD2-18 vs. wt

genotype	nr.	repeat	plant height, cm	number of leaves	leaf biomass, g	stem biomass, g
At-CycD2-18	1	1	11.2	15	9.31	4.28
At-CycD2-18	2	1	12.4	12	10.1	4.6
At-CycD2-18	3	1	10.2	11	8.77	3.78
At-CycD2-18	4	1	10.8	17	13.5	5.81
At-CycD2-18	5	1	10.5	13	10.3	4.45
At-CycD2-18	6	1	10.5	29	8.99	4.22
At-CycD2-18	7	1	11	19	10.2	4.56
At-CycD2-18	8	1	8.4	12	9.44	3.28
At-CycD2-18	9	1	12	11	10.9	5.19
At-CycD2-18	10	1	9.5	19	10.9	4.02
At-CycD2-18	11	1	10	10	8.22	3.96
At-CycD2-18	12	1	10.6	10	7.7	3.82
At-CycD2-18	13	1	10.8	9	9.57	4.19
At-CycD2-18	14	1	8.5	13	8.06	3.23
At-CycD2-18	15	1	7	10	5.44	2.28
At-CycD2-18	16	2	15.4	17	17.3	9.37
At-CycD2-18	17	2	9.1	11	9.12	3.7
At-CycD2-18	18	2	15	16	14.5	7.11
At-CycD2-18	19	2	19.2	19	13.6	7.17
At-CycD2-18	20	2	19	19	16.5	7.78
At-CycD2-18	21	2	14	18	11.6	5.63
At-CycD2-18	22	2	13.8	10	8.92	4.23
At-CycD2-18	23	2	11.8	13	7.81	3.19
At-CycD2-18	24	2	16.5	14	10.4	5.38
At-CycD2-18	25	2	12	23	21.6	11.4
At-CycD2-18	26	2	15.2	19	13.4	6.83
At-CycD2-18	27	2	11.3	18	9.35	3.38
At-CycD2-18	28	2	15	22	15.9	7.33
At-CycD2-18	29	2	13.5	24	15.2	7.03
At-CycD2-18	30	2	14.6	16	14.4	6.82
At-CycD2-18	31	3	15.5	13	9.01	4.27
At-CycD2-18	32	3	16.7	13	7.95	3.43
At-CycD2-18	33	3	9	9	5.23	1.93
At-CycD2-18	34	3	16.6	21	14.8	7.07
At-CycD2-18	35	3	17.4	22	16.2	8.06
At-CycD2-18	36	3	20.6	23	17.7	10.4
At-CycD2-18	37	3	14.5	19	10.6	4.92
At-CycD2-18	38	3	15	15	9.61	4.24
At-CycD2-18	39	3	14.6	11	7.03	2.95
At-CycD2-18	40	3	16	13	10.7	5.55
At-CycD2-18	41	3	16.3	18	8.97	4.51
At-CycD2-18	42	3	13.2	18	9.43	4.78
At-CycD2-18	43	3	13.3	17	10.4	4.26
At-CycD2-18	44	3	15.8	14	10.7	4.73
At-CycD2-18	45	3	13	17	14.1	6.09

genotype	nr.	repeat	plant height, cm	number of leaves	leaf biomass, g	stem biomass, g
wt	1	1	12.7	18	10.1	5.93
wt	2	1	12.2	22	10.6	5.63
wt	3	1	13.0	25	11.5	6.63
wt	4	1	12.0	21	9.3	5.26
wt	5	1	12.5	20	10.8	5.3
wt	6	1	12.2	22	10.9	5.61
wt	7	1	13.8	21	10.5	5.75
wt	8	1	13.2	24	11.6	6.2
wt	9	1	13.5	20	10.5	5.2
wt	10	1	13.2	21	10.8	5.88
wt	11	1	12.3	23	9.84	5.6
wt	12	1	11	19	8.44	4.19
wt	13	1	13	26	11.3	5.95
wt	14	1	13.6	23	10.4	5.65
wt	15	1	7.8	11	3.89	1.74
wt	16	2	16.3	30	13.4	7.98
wt	17	2	20.6	30	17.9	11.1
wt	18	2	25.5	41	23.5	15.2
wt	19	2	22.7	39	23.3	13.5
wt	20	2	20.7	35	18.3	10.1
wt	21	2	22.0	36	21.6	13.4
wt	22	2	22.3	31	15.8	10.1
wt	23	2	21	33	19	10.4
wt	24	2	17.2	28	12.7	6.7
wt	25	2	17.3	30	16.8	9.12
wt	26	2	21	28	15.3	8.52
wt	27	2	19.6	34	18.8	11
wt	28	2	17.1	28	13.6	7.44
wt	29	2	17.6	32	14.9	7.69
wt	30	2	19.8	37	17.2	9.84
wt	31	3	15.5	24	9.54	4.78
wt	32	3	18.5	31	12.9	6.55
wt	33	3	19.5	23	11	5.77
wt	34	3	17.0	26	9.19	4.9
wt	35	3	16.3	26	10.2	5.05
wt	36	3	13.5	20	7.88	3.58
wt	37	3	22	31	15.2	8.22
wt	38	3	17	30	13	6.06
wt	39	3	17.7	24	11.2	5.76
wt	40	3	17	26	10.7	5.33
wt	41	3	17.3	25	9.42	5.12
wt	42	3	17.7	33	16.9	8.24
wt	43	3	16.5	22	10.3	4.92
wt	44	3	18	26	12.4	6.29
wt	45	3	15	27	9.46	4.75

At-CycD2-21 vs. wt

genotype	nr.	repeat	plant height, cm	number of leaves	leaf biomass, g	stem biomass, g
At-CycD2-21	1	1	12.5	21	17.3	7.8
At-CycD2-21	2	1	14	24	16.2	6.8
At-CycD2-21	3	1	14.2	27	18.7	10.7
At-CycD2-21	4	1	14.6	23	19.1	8.38
At-CycD2-21	5	1	11.5	17	14.3	5.84
At-CycD2-21	6	1	13.3	23	14.2	6.43
At-CycD2-21	7	1	12.5	19	17	7.36
At-CycD2-21	8	1	12	23	16.2	6.25
At-CycD2-21	9	1	13.1	22	14.5	6.4
At-CycD2-21	10	1	14.3	25	14.7	5.55
At-CycD2-21	11	1	12.7	24	12	5.73
At-CycD2-21	12	1	13.7	23	15.2	6.98
At-CycD2-21	13	1	11.3	19	14.1	4.85
At-CycD2-21	14	1	13.1	16	15	6.57
At-CycD2-21	15	1	12.1	18	15.3	6.61
At-CycD2-21	16	2	19.6	13	22.3	10.4
At-CycD2-21	17	2	21.3	17	15	7.62
At-CycD2-21	18	2	18.6	12	9.7	4.45
At-CycD2-21	19	2	19.7	14	14.2	7.56
At-CycD2-21	20	2	16.3	18	16.8	8.06
At-CycD2-21	21	2	18.4	12	11.7	6.24
At-CycD2-21	22	2	19.3	27	23	15
At-CycD2-21	23	2	19.7	19	15.6	7.93
At-CycD2-21	24	2	16.1	13	10.5	4.4
At-CycD2-21	25	2	21.8	15	17	9.42
At-CycD2-21	26	2	18.1	16	13.3	6.52
At-CycD2-21	27	2	20.8	28	12.9	7.38
At-CycD2-21	28	2	14.7	22	17.6	8.19
At-CycD2-21	29	2	22.6	25	18.9	10.4
At-CycD2-21	30	2	25	21	22.6	12.1
At-CycD2-21	31	3	15.2	26	14.39	5.48
At-CycD2-21	32	3	17.3	18	14.00	4.74
At-CycD2-21	33	3	13.3	15	10.34	3.53
At-CycD2-21	34	3	20.4	13	13.20	6.61
At-CycD2-21	35	3	17.2	15	11.92	4.88
At-CycD2-21	36	3	14.4	14	5.87	3.12
At-CycD2-21	37	3	17.9	12	15.19	6.36
At-CycD2-21	38	3	19.2	14	12.68	5.77
At-CycD2-21	39	3	21.3	14	18.00	7.83
At-CycD2-21	40	3	18.9	13	12.40	5.90
At-CycD2-21	41	3	22.3	19	15.79	8.51
At-CycD2-21	42	3	22.4	13	16.35	9.16
At-CycD2-21	43	3	16.8	14	9.72	4.44
At-CycD2-21	44	3	18.9	27	14.50	7.37
At-CycD2-21	45	3	13.2	11	6.37	3.20

genotype	nr.	repeat	plant height, cm	number of leaves	leaf biomass, g	stem biomass, g
wt	1	1	11.5	25	8.6	4.12
wt	2	1	10.4	21	8.2	3.75
wt	3	1	12.4	27	10.9	5.29
wt	4	1	9.3	17	4.84	2.06
wt	5	1	9.8	14	4.8	2.14
wt	6	1	11.7	21	7.99	3.82
wt	7	1	10.6	22	7.39	3.22
wt	8	1	8.2	11	3.39	1.79
wt	9	1	10	18	6.18	2.81
wt	10	1	10.7	20	8.71	4.1
wt	11	1	11.4	24	9.06	4.42
wt	12	1	11.8	26	11.7	5.53
wt	13	1	12.5	26	11.5	5.36
wt	14	1	12.7	29	12.4	6.09
wt	15	1	11.7	23	11.2	5.41
wt	16	2	24.4	40	26.7	16.2
wt	17	2	26.4	43	20.7	13.6
wt	18	2	26.6	53	22.9	15.8
wt	19	2	27.6	31	15.7	10.3
wt	20	2	27.4	49	22.4	15.3
wt	21	2	28.7	54	29.5	21.3
wt	22	2	28.4	38	22.8	14.7
wt	23	2	27.5	40	22.4	15.4
wt	24	2	29	38	24.5	17.6
wt	25	2	28.4	42	23.9	17
wt	26	2	28.4	44	23	15.9
wt	27	2	26.2	40	26.1	16
wt	28	2	26.3	44	22	12.6
wt	29	2	27.6	38	21.2	14.4
wt	30	2	22.2	37	18.2	12
wt	31	3	28.1	42	24.69	14.72
wt	32	3	28.7	40	23.24	13.80
wt	33	3	29.1	44	26.51	15.16
wt	34	3	28.2	44	24.87	15.24
wt	35	3	32.2	44	26.96	16.69
wt	36	3	25.3	38	18.89	11.44
wt	37	3	24.9	35	19.92	10.58
wt	38	3	26.3	33	21.16	12.11
wt	39	3	28.6	40	23.19	13.92
wt	40	3	28.4	29	16.99	9.74
wt	41	3	28.3	36	20.90	12.88
wt	42	3	26.9	37	18.51	10.26
wt	43	3	28.6	44	23.34	13.78
wt	44	3	27.7	44	25.88	13.95
wt	45	3	27.6	35	19.54	10.89

At-CycD2-24 vs. wt

genotype	nr.	repeat	plant height, cm	number of leaves	leaf biomass, g	stem biomass, g
At-CycD2-24	1	1	7.2	10	7.53	3.7
At-CycD2-24	2	1	10	18	7.25	3.78
At-CycD2-24	3	1	7.2	19	7.47	2.86
At-CycD2-24	4	1	7	14	8.48	3.49
At-CycD2-24	5	1	8.7	23	10.9	5.81
At-CycD2-24	6	1	11.4	23	13.3	5.99
At-CycD2-24	7	1	11.1	20	13.6	6.2
At-CycD2-24	8	1	6.5	15	10.3	3.39
At-CycD2-24	9	1	8.2	20	8.47	3.78
At-CycD2-24	10	1	10.8	20	9.84	5.2
At-CycD2-24	11	1	9.1	20	7.32	3.36
At-CycD2-24	12	1	11.8	23	15.7	6.57
At-CycD2-24	13	1	11.5	28	13.2	7.72
At-CycD2-24	14	1	10.5	24	10.1	5.48
At-CycD2-24	15	1	8.5	23	8.3	4
At-CycD2-24	16	2	16	29	24.7	12.7
At-CycD2-24	17	2	18	20	21.6	11.3
At-CycD2-24	18	2	20.6	18	19.9	10.3
At-CycD2-24	19	2	17.6	32	15.1	10
At-CycD2-24	20	2	22.9	36	25.3	18.2
At-CycD2-24	21	2	19.3	34	21.3	12.6
At-CycD2-24	22	2	21.2	32	28.8	16.5
At-CycD2-24	23	2	21.4	28	22.4	13.6
At-CycD2-24	24	2	18.2	30	21.2	11
At-CycD2-24	25	2	19.4	28	24.4	13.6
At-CycD2-24	26	2	16.3	27	16.9	9.92
At-CycD2-24	27	2	20.2	27	22.8	10.5
At-CycD2-24	28	2	19	28	26.7	13.4
At-CycD2-24	29	2	20.7	25	26	13.9
At-CycD2-24	30	2	20.5	33	22.3	15
At-CycD2-24	31	3	7.3	23	11.7	5.52
At-CycD2-24	32	3	8.8	21	11.3	5.45
At-CycD2-24	33	3	8	18	10.5	4.32
At-CycD2-24	34	3	9	15	7.56	3.07
At-CycD2-24	35	3	7.7	20	11.9	4.56
At-CycD2-24	36	3	6.5	11	9.6	3.59
At-CycD2-24	37	3	11	31	10.7	5.31
At-CycD2-24	38	3	8.5	12	3.5	1.96
At-CycD2-24	39	3	6.8	17	8.41	3.23
At-CycD2-24	40	3	8.3	18	9.56	3.82
At-CycD2-24	41	3	6.3	9	2.68	1.39
At-CycD2-24	42	3	8	12	8.46	2.93
At-CycD2-24	43	3	4	16	9.75	2.89
At-CycD2-24	44	3	8.2	20	8.87	4.14
At-CycD2-24	45	3	6	16	5.97	3.06

genotype	nr.	repeat	plant height, cm	number of leaves	leaf biomass, g	stem biomass, g
wt	1	1	11.5	25	8.6	4.12
wt	2	1	10.4	21	8.2	3.75
wt	3	1	12.4	27	10.9	5.29
wt	4	1	9.3	17	4.84	2.06
wt	5	1	9.8	14	4.8	2.14
wt	6	1	11.7	21	7.99	3.82
wt	7	1	10.6	22	7.39	3.22
wt	8	1	8.2	11	3.39	1.79
wt	9	1	10	18	6.18	2.81
wt	10	1	10.7	20	8.71	4.1
wt	11	1	11.4	24	9.06	4.42
wt	12	1	11.8	26	11.7	5.53
wt	13	1	12.5	26	11.5	5.36
wt	14	1	12.7	29	12.4	6.09
wt	15	1	11.7	23	11.2	5.41
wt	16	2	24.4	40	26.7	16.2
wt	17	2	26.4	43	20.7	13.6
wt	18	2	26.6	53	22.9	15.8
wt	19	2	27.6	31	15.7	10.3
wt	20	2	27.4	49	22.4	15.3
wt	21	2	28.7	54	29.5	21.3
wt	22	2	28.4	38	22.8	14.7
wt	23	2	27.5	40	22.4	15.4
wt	24	2	29	38	24.5	17.6
wt	25	2	28.4	42	23.9	17
wt	26	2	28.4	44	23	15.9
wt	27	2	26.2	40	26.1	16
wt	28	2	26.3	44	22	12.6
wt	29	2	27.6	38	21.2	14.4
wt	30	2	22.2	37	18.2	12
wt	31	3	16.2	24	13	6.58
wt	32	3	11.8	23	10.7	6.32
wt	33	3	16.0	22	11.3	5.74
wt	34	3	12.5	19	9.35	4.29
wt	35	3	17.0	22	10.6	5.76
wt	36	3	16.6	24	11.1	4.66
wt	37	3	18	25	15.8	5.18
wt	38	3	16.2	19	10.5	5.07
wt	39	3	13	18	11.6	4.86
wt	40	3	17.5	22	13.7	6.72
wt	41	3	18.3	27	12	4.66
wt	42	3	18.2	19	10.9	6.41
wt	43	3	15	25	14.2	6.62
wt	44	3	15.5	19	11.1	5.23
wt	45	3	14.3	23	12.8	5.5

At-CycD2-25 vs. wt

genotype	nr.	repeat	plant height, cm	number of leaves	leaf biomass, g	stem biomass, g
At-CycD2-25	1	1	16.5	24	19	8.84
At-CycD2-25	2	1	15	21	14.4	6.95
At-CycD2-25	3	1	16.3	23	12.6	6.94
At-CycD2-25	4	1	15	23	14.4	6.8
At-CycD2-25	5	1	15.3	21	12.1	6.52
At-CycD2-25	6	1	15.8	23	13.4	6.8
At-CycD2-25	7	1	15.2	27	21	10.3
At-CycD2-25	8	1	14.8	23	16.6	7.65
At-CycD2-25	9	1	16.3	21	14.5	7.45
At-CycD2-25	10	1	15.6	23	15.3	7.61
At-CycD2-25	11	1	16.8	20	15.8	8.12
At-CycD2-25	12	1	16.4	25	16.8	8
At-CycD2-25	13	1	16.4	26	20.2	9.69
At-CycD2-25	14	1	17.5	23	13.2	7.28
At-CycD2-25	15	1	13.7	20	12.9	6.25
At-CycD2-25	16	2	15	20	11.7	6.41
At-CycD2-25	17	2	14.8	25	10.2	6.11
At-CycD2-25	18	2	17.6	30	16.6	9.92
At-CycD2-25	19	2	14.8	24	10.4	6.54
At-CycD2-25	20	2	14	28	11.5	6.85
At-CycD2-25	21	2	16.8	31	15.7	9.14
At-CycD2-25	22	2	16	32	15.5	9.72
At-CycD2-25	23	2	16.7	29	14	8.64
At-CycD2-25	24	2	14.4	26	12.6	6.76
At-CycD2-25	25	2	14.4	25	13	7.14
At-CycD2-25	26	2	14.3	24	10.4	5.97
At-CycD2-25	27	2	13.8	26	11.3	6.33
At-CycD2-25	28	2	15	27	14.7	8.45
At-CycD2-25	29	2	14.2	28	14.1	7.76
At-CycD2-25	30	2	16	28	15.8	9.1
At-CycD2-25	31	3	11	18	10.9	5.06
At-CycD2-25	32	3	11.7	17	11	5.07
At-CycD2-25	33	3	11	19	10.2	4.72
At-CycD2-25	34	3	12.5	17	10.4	5.03
At-CycD2-25	35	3	10.5	24	11.3	4.92
At-CycD2-25	36	3	11.5	23	10.9	5.21
At-CycD2-25	37	3	10.2	18	9.91	4.63
At-CycD2-25	38	3	10.3	14	8.69	4.01
At-CycD2-25	39	3	11.2	21	10.4	4.82
At-CycD2-25	40	3	10.2	22	10.4	4.66
At-CycD2-25	41	3	9.8	18	7.94	3.68
At-CycD2-25	42	3	10	20	9.1	4.08
At-CycD2-25	43	3	10.8	20	9.94	4.73
At-CycD2-25	44	3	9.7	22	12	4.83
At-CycD2-25	45	3	10.2	20	9.26	4.15

genotype	nr.	repeat	plant height, cm	number of leaves	leaf biomass, g	stem biomass, g
wt	1	1	16.2	24	13	6.58
wt	2	1	11.8	23	10.7	6.32
wt	3	1	16.0	22	11.3	5.74
wt	4	1	12.5	19	9.35	4.29
wt	5	1	17.0	22	10.6	5.76
wt	6	1	16.6	24	11.1	4.66
wt	7	1	18	25	15.8	5.18
wt	8	1	16.2	19	10.5	5.07
wt	9	1	13	18	11.6	4.86
wt	10	1	17.5	22	13.7	6.72
wt	11	1	18.3	27	12	4.66
wt	12	1	18.2	19	10.9	6.41
wt	13	1	15	25	14.2	6.62
wt	14	1	15.5	19	11.1	5.23
wt	15	1	14.3	23	12.8	5.5
wt	16	2	11.5	25	8.6	4.12
wt	17	2	10.4	21	8.2	3.75
wt	18	2	12.4	27	10.9	5.29
wt	19	2	9.3	17	4.84	2.06
wt	20	2	9.8	14	4.8	2.14
wt	21	2	11.7	21	7.99	3.82
wt	22	2	10.6	22	7.39	3.22
wt	23	2	8.2	11	3.39	1.79
wt	24	2	10	18	6.18	2.81
wt	25	2	10.7	20	8.71	4.1
wt	26	2	11.4	24	9.06	4.42
wt	27	2	11.8	26	11.7	5.53
wt	28	2	12.5	26	11.5	5.36
wt	29	2	12.7	29	12.4	6.09
wt	30	2	11.7	23	11.2	5.41
wt	31	3	17.3	40	15.7	10.2
wt	32	3	16	34	17.5	9.86
wt	33	3	16.3	30	16.2	9.08
wt	34	3	12.2	26	11.8	5.38
wt	35	3	11.8	20	13.6	4.61
wt	36	3	12.2	19	12.8	5.06
wt	37	3	11.8	19	9.51	4.41
wt	38	3	12	26	10.3	4.87
wt	39	3	10.9	20	12.3	5.17
wt	40	3	13.2	21	13.3	5.72
wt	41	3	12	24	11.3	5.01
wt	42	3	10	22	10.5	4.22
wt	43	3	11.8	24	11.6	5.04
wt	44	3	10	26	11	4.44
wt	45	3	10.5	26	11.3	4.51

Table S4. Phenotypic characterization of transgenic *N. benthamiana* lines expressing the At-CycD2 gene. Non-transgenic and T_1 progeny of At-CycD2-6, At-CycD2-14, At-CycD2-15 and At-CycD2-25 transgenic plants were grown in greenhouse for 4 weeks and assayed for the plant height, number of leaves and above ground biomass characteristics. The phenotypic parameters are expressed as a percent relative to wild type. Data for three replications are shown (n=45). Each replication included 15 plants per transgenic line or wild type non-transformed control. Values represent the means with standard deviation. The unpaired T-test using SigmaStat statistic software was applied to determine differences among the mean values.

	plant height		number of leaves	
	transgenic plant	wt control	transgenic plant	wt control
**At-CycD2-6**	93.96 ± 11.63	100.00 ± 10.80	101.83 ± 17.68	100.00 ± 14.33
**At-CycD2-11**	102.81 ± 9.53	100.00 ± 11.06	87.79 ± 13.26	100.00 ± 12.93
**At-CycD2-12**	99.13 ± 15.11	100.00 ± 9.09	86.06 ± 21.15	100.00 ± 16.31
**At-CycD2-14**	88.36 ± 17.02	100.00 ± 11.69	104.99 ± 21.15	100.00 ± 15.44
**At-CycD2-15**	90.47 ± 13.94	100.00 ± 12.18	123.11 ± 22.02	100.00 ± 16.35
**At-CycD2-16**	100.91 ± 14.65	100.00 ± 9.09	82.90 ± 28.01	100.00 ± 16.31
**At-CycD2-17**	91.24 ± 9.56	100.00 ± 10.44	76.28 ± 16.94	100.00 ± 13.68
**At-CycD2-18**	80.70 ± 14.72	100.00 ± 11.76	60.26 ± 18.92	100.00 ± 14.16
**At-CycD2-21**	84.85 ± 26.07	100.00 ± 8.33	61.26 ± 30.60	100.00 ± 16.90
**At-CycD2-24**	68.35 ± 19.20	100.00 ± 10.59	79.50 ± 22.01	100.00 ± 16.95
**At-CycD2-25**	107.98 ± 23.82	100.00 ± 14.19	101.91 ± 22.38	100.00 ± 19.83

	leaf biomass		stem biomass	
	transgenic plant	wt control	transgenic plant	wt control
**At-CycD2-6**	134.03 ± 27.18	100.00 ± 15.81	124.87 ± 26.55	100.00 ± 16.97
**At-CycD2-11**	107.32 ± 18.91	100.00 ± 17.14	110.46 ± 21.76	100.00 ± 19.14
**At-CycD2-12**	109.14 ± 29.01	100.00 ± 21.70	109.44 ± 32.92	100.00 ± 22.62
**At-CycD2-14**	135.39 ± 28.39	100.00 ± 18.70	119.78 ± 33.67	100.00 ± 20.80
**At-CycD2-15**	142.59 ± 29.43	100.00 ± 19.41	140.38 ± 25.91	100.00 ± 21.84
**At-CycD2-16**	110.66 ± 38.13	100.00 ± 21.70	98.02 ± 34.77	100.00 ± 22.62
**At-CycD2-17**	79.59 ± 18.88	100.00 ± 17.19	75.87 ± 18.97	100.00 ± 20.18
**At-CycD2-18**	88.73 ± 18.89	100.00 ± 19.45	76.80 ± 28.18	100.00 ± 21.97
**At-CycD2-21**	103.95 ± 60.69	100.00 ± 21.74	90.12 ± 62.48	100.00 ± 23.50
**At-CycD2-24**	97.27 ± 30.72	100.00 ± 21.75	89.88 ± 34.02	100.00 ± 23.18
**At-CycD2-25**	122.15 ± 37.39	100.00 ± 22.64	136.33 ± 51.83	100.00 ± 28.97
